# Evolution, Expression Patterns, and Distribution of Novel Ribbon Worm Predatory and Defensive Toxins

**DOI:** 10.1093/molbev/msac096

**Published:** 2022-05-05

**Authors:** Aida Verdes, Sergi Taboada, Brett R. Hamilton, Eivind A.B. Undheim, Gabriel G. Sonoda, Sonia C.S. Andrade, Esperanza Morato, Ana Isabel Marina, César A. Cárdenas, Ana Riesgo

**Affiliations:** Department of Biodiversity and Evolutionary Biology, Museo Nacional de Ciencias Naturales (MNCN), CSIC, Madrid, Spain; Department of Life Sciences, Natural History Museum, London, UK; Department of Life Sciences, Natural History Museum, London, UK; Departament of Biodiversity, Ecology, and Evolution, Universidad Complutense de Madrid, Madrid, Spain; Centre for Advanced Imaging, The University of Queensland, Brisbane, QLD, Australia; Centre for Microscopy and Microanalysis, The University of Queensland, Brisbane, QLD, Australia; Centre for Advanced Imaging, The University of Queensland, Brisbane, QLD, Australia; Centre for Ecological and Evolutionary Synthesis, Department of Biosciences, University of Oslo, PO Box 1066, Blindern, 0316 Oslo, Norway; Institute for Molecular Bioscience, The University of Queensland, Brisbane, QLD, Australia; Departmento de Genética e Biología Evolutiva, University of Sao Paulo, Sao Paulo, Brazil; Departmento de Genética e Biología Evolutiva, University of Sao Paulo, Sao Paulo, Brazil; CBMSO Protein Chemistry Facility, Centro de Biología Molecular Severo Ochoa, Consejo Superior de Investigaciones Científicas, Universidad Autónoma de Madrid, Madrid, Spain; CBMSO Protein Chemistry Facility, Centro de Biología Molecular Severo Ochoa, Consejo Superior de Investigaciones Científicas, Universidad Autónoma de Madrid, Madrid, Spain; Departamento Científico, Instituto Antártico Chileno, Punta Arenas, Chile; Millennium Institute Biodiversity of Antarctic and Subantarctic Ecosystems (BASE), Santiago, Chile; Department of Biodiversity and Evolutionary Biology, Museo Nacional de Ciencias Naturales (MNCN), CSIC, Madrid, Spain; Department of Life Sciences, Natural History Museum, London, UK

**Keywords:** venom, toxins, RNAseq, ribbon worm, mass spectrometry imaging, proteomics

## Abstract

Ribbon worms are active predators that use an eversible proboscis to inject venom into their prey and defend themselves with toxic epidermal secretions. Previous work on nemertean venom has largely focused on just a few species and has not investigated the different predatory and defensive secretions in detail. Consequently, our understanding of the composition and evolution of ribbon worm venoms is still very limited. Here, we present a comparative study of nemertean venom combining RNA-seq differential gene expression analyses of venom-producing tissues, tandem mass spectrometry-based proteomics of toxic secretions, and mass spectrometry imaging of proboscis sections, to shed light onto the composition and evolution of predatory and defensive toxic secretions in *Antarctonemertes valida*. Our analyses reveal a wide diversity of putative defensive and predatory toxins with tissue-specific gene expression patterns and restricted distributions to the mucus and proboscis proteomes respectively, suggesting that ribbon worms produce distinct toxin cocktails for predation and defense. Our results also highlight the presence of numerous lineage-specific toxins, indicating that venom evolution is highly divergent across nemerteans, producing toxin cocktails that might be finely tuned to subdue different prey. Our data also suggest that the hoplonemertean proboscis is a highly specialized predatory organ that seems to be involved in a variety of biological functions besides predation, including secretion and sensory perception. Overall, our results advance our knowledge into the diversity and evolution of nemertean venoms and highlight the importance of combining different types of data to characterize toxin composition in understudied venomous organisms.

## Introduction

Animal venoms are key evolutionary adaptations that have evolved convergently in more than 100 different animal lineages to assist in vital functions such as defense, predation, and competition (Casewell et al. 2013; Schendel et al. 2019). Venoms are complex toxic biological secretions produced by one animal and delivered to another animal through the infliction of a wound (Fry et al. 2009; Casewell et al. 2013). They are comprised of a mixture of bioactive compounds denominated toxins that disrupt the normal physiology of their targets, affecting fundamental processes such as blood coagulation and homeostasis, and interfering with central pathways in the cardiovascular and neuromuscular systems (Calvete et al. 2009). Most toxins are proteins and peptides thought to have evolved through duplication, and recruitment of genes that play normal physiological roles into venom, where they acquire novel functions as toxins (Casewell et al. 2013; Jenner et al. 2019). Despite the complexity of venoms, there is a remarkable degree of convergence in the molecular structures and targets of most toxins (Fry et al. 2009), making venomous organisms great model systems to investigate biological questions in areas as diverse as molecular evolution, functional convergence, and drug discovery. However, the processes underlying toxin and venom evolution remain poorly understood, in particular for many invertebrate groups. Nevertheless, the advancement of sequencing and analytical techniques has expanded research to traditionally neglected taxa, shedding light into venom biology of species that were previously challenging to work with, like mammals (Casewell et al. 2019; Nekaris et al. 2020) or small invertebrates (Gorson et al. 2015; Robinson et al. 2018; Jenner et al. 2019; von Reumont et al. 2020; Walker et al. 2021). This increase in the taxonomic coverage of the venomous taxa being investigated is revealing a high genetic and functional diversity of venom compounds across taxa (Madio et al. 2018; Robinson et al. 2018; Giorgianni et al. 2020), as well as novel mechanisms of venom evolution (Sachkova et al. 2020; Undheim and Jenner 2021), challenging traditional views in venom research. Still, many invertebrate groups such as ribbon worms (Nemertea)—venomous active predators that use toxins for defense and predation—remain understudied.

**Fig. 1.**
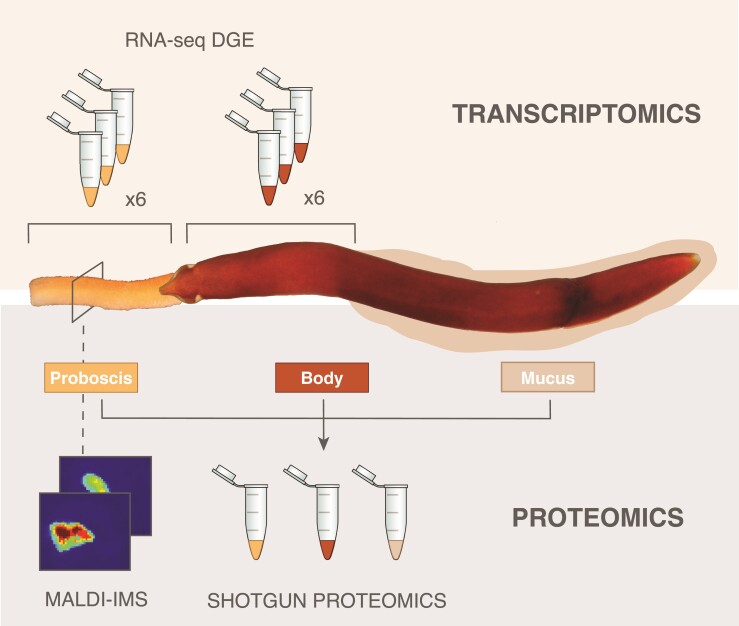
Overview of experimental approach. We generated 12 RNA-seq libraries from the proboscis and body tissue of six *Antarctonemertes valida* individuals (biological replicates) for DGE analyses. We also performed shotgun proteomic analyses of a complete specimen, defensive mucus, and three proboscis samples. In addition, we used MALDI-IMS to look at the peptide distribution in a transversal section of a proboscis. We combined all data to identify putative novel nemertean toxins for defense and predation.

Ribbon worms, or nemerteans, are a phylum of unsegmented worms with more than 1,300 species described to date (Kajihara et al. 2008). Most species are active predators that use an eversible proboscis to inject toxins into their prey and defend themselves with a toxic epidermal mucous secretion (fig. 1). Nemerteans do not have distinct multicellular glands—instead, toxins are secreted by cells lining the body wall and proboscis epithelia. Although all nemerteans have an eversible proboscis, there are important morphological differences among the three groups in which the phylum is currently divided: Palaeonemertea, Pilidiophora, and Hoplonemertea (Andrade et al. 2014). The hoplonemertean proboscis is armed with a calcareous stylet used to stab prey and thought to inject toxins, whereas Palaeonemertea and Pilidiophora lack stylets, but contain rod-shaped secretory bodies hypothesized to puncture the prey body wall facilitating envenomation (Stricker and Cloney 1983). Previous work on nemertean venom has largely focused on just a few species, identifying a handful of nonproteinaceous toxins, such as anabaseine and tetrodotoxin (Ali et al. 1990), and some cytolytic and neurotoxic peptides including parborlysin, cytotoxin A-III, and neurotoxin B-IV (Kem 1976). Only a couple of studies have surveyed nemertean toxin gene diversity using high-throughput technologies, discovering a much greater diversity of toxin-like genes than previously documented (Whelan et al. 2014; von Reumont et al. 2020). Moreover, a novel family of cysteine knot peptides with potent activity on sodium channels was recently isolated from the defensive mucus of *Lineus longissimus* (Gunnerus 1770) and later patented as a pesticide (Jacobsson et al. 2018). These findings suggest there is a significant undiscovered diversity of ribbon worm toxins.

The way toxins evolve, mainly by duplication and recruitment of genes with nontoxic physiological functions into a venom-producing tissue, makes it challenging to identify putative novel toxins relying on transcriptome data exclusively because it often leads to false positives (Smith and Undheim 2018). This is even more problematic with venomous organisms that do not have distinct multicellular venom glands like cnidarians or ribbon worms, because in these cases obtaining crude venom is usually also challenging. One way to minimize these difficulties and identify putative toxins more reliably, is to use RNA-seq differential gene expression (DGE) analyses to compare gene expression levels in venom-producing tissues with those of an alternative tissue, which might help distinguish toxins from their nontoxin homologs based on statistically higher expression levels in the venom-producing tissue (Modica et al. 2015; Macrander et al. 2016). In addition, mass spectrometry imaging (MSI), which is a nontargeted approach that allows to explore the spatial distribution of peptides without prior knowledge of their identities (Caldwell and Caprioli 2005; Maier et al. 2013; Walker et al. 2020), is increasingly being used to correlate the distribution of toxins with specific venom-producing tissue regions in a variety of taxa including cnidarians, centipedes, and snakes (Undheim, Grimm, 2015; Undheim, Hamilton, 2015; Mitchell et al. 2017; Madio et al. 2018; Hamilton et al. 2020). Therefore, the combination of RNA-seq DGE analyses with MSI is a powerful approach that allows to associate individual toxins with specific histological features (e.g., secretory cells), providing even more robust toxin predictions and reducing the probability of false positives.

In this study, we present the first comparative analysis of ribbon worm venom combining RNA-seq DGE of venom-producing tissues, tandem mass spectrometry-based proteomics (MS/MS) of toxic secretions, and MSI of proboscis tissue sections to shed light onto the composition and evolution of predatory and defensive toxic secretions in the Antarctic hoplonemertean *Antarctonemertes valida* (Bürger 1983) (fig. 1). Our analyses provide a comprehensive picture of ribbon worm venom composition and reveal a wide diversity of putative defensive and predatory toxins that are differentially expressed and found in the mucus and proboscis proteomes respectively. Our results advance our knowledge into the diversity and evolution of nemertean venoms and highlight the importance of combining different lines of evidence to explore toxin composition in understudied venomous organisms.

## Materials and Methods

### Sample Collection, RNA Extraction, and Sequencing

We collected samples of the species *A. valida* and *A. riesgoae* (Taboada et al. 2013) by hand during an Antarctic campaign in January and February 2016. Sampling was performed at low tide in the intertidal, in sheltered, rocky beaches, collecting the organisms attached to rocks and algae. For *A. valida*, we collected a total of six individuals, three from each of two sampling sites: Crater 70 at Deception Island, South Shetland Islands (62°55.507′S; 60°37.892′W); and Esperanza Bay, Western Antarctic Peninsula (63°23.782′S; 56° 9.780′W). For *A. riesgoae*, we collected one individual from Gabriel de Castilla Spanish Antarctic Base at Deception Island, South Shetland Islands (62°58.569′S; 60°40.513′W). We preserved the specimens in RNA*later* (Life Technologies) immediately after collection, stored for 24 h at 4 °C, replaced the RNA*later* once, and then stored at −80 °C until further processing. Three additional specimens of *A. valida* were collected by hand at Fildes Bay, King George Island, South Shetland Islands (62°11'55”S; 58°56'59”W) in January 2019. Before preservation, specimens were induced to produce defensive epidermal mucus by grabbing them with a pair of forceps against a tube wall without water. Mucus produced by each of the three specimens and the three specimens were immediately flash frozen and stored at −20 °C for proteomic analyses.

Before RNA extraction, the proboscis of the *A. valida* individuals was dissected and total RNA was extracted separately for the proboscis and body using a standard Trizol-based method with TRI Reagent (Life Sciences) following the manufacturer's instructions (fig. 1). For *A. riesgoae*, the whole individual including the proboscis was used for RNA extraction. Quantity and quality of RNA were measured with Qubit and TapeStation and subsequent mRNA purification was performed with Dynabeads mRNA Purification Kit (Invitrogen) following the manufacturer's protocol. cDNA libraries were constructed with the ScriptSeq v2 kit (Illumina) and sequenced in a NextSeq 500, at 150 bp paired end read length at the Sequencing Facilities of the Natural History Museum of London.

### Sequence Processing and De Novo Transcriptome Assembly

Transcriptomic raw reads were cleaned using Trimmomatic 0.33 (Bolger et al. 2014) with the following settings: ILLUMINACLIP:/Adapters.fa:2:30:10 LEADING:3 TRAILING:3 SLIDINGWINDOW:4:20 MINLEN:30 where the Adapters.fa file was substituted for the appropriate adapters for each library. Raw data were deposited in the NCBI SRA database and corresponding accession numbers can be found in (supplementary file S1 and table S1, Supplementary Material online). To ensure complete removal of adapter and low-quality sequence data, sequence quality was assessed using FastQC (Andrews 2010) before and after trimming. A de novo reference transcriptome was assembled for each species (for *A. valida* using six RNA-seq libraries and for *A. riesgoae* using one RNA-seq library) with the Trinity version 2.4.4 (Grabherr et al. 2011). The assembly of *A. valida* contained 388 Mb and 681,621 transcripts with an N50 of 696. The assembly of *A. riesgoae* comprised a total of 68.2 Mb and 100,829 transcripts, and an N50 of 672 (supplementary file S1 and table S2, Supplementary Material online). In addition, the reference transcriptome of *A. valida* was translated to all six possible open reading frames longer than 30 amino acids using TransDecoder v5.0.2 (Grabherr et al. 2013) and EMBOSS Getorf (Rice et al. 2000) to generate the predicted proteome dataset.

### Differential Expression Analysis and Functional Annotation

A Differential Expression Analysis (DGE) to identify genes overexpressed or upregulated in the proboscis tissue was performed with the Trinity module, which incorporates RSEM and edgeR (Robinson et al. 2009; Garber et al. 2011; Li and Dewey 2011). Individual libraries corresponding to proboscis and posterior end tissue of each replicate were mapped to the reference transcriptome. The software edgeR was implemented with a *P*-value cut-off for false discovery rate (FDR) of 0.001, and a min abs(log2[*a*/*b*]) change of 2 (therefore, minimally, 4-fold change).

We performed automated annotation using Diamond (Buchfink et al. 2014) against the UniProtKB database (The UniProt Consortium 2015) accessed in 2021, with a cut-off *e*-value of 1 × 10^−5^ (supplementary file S2, Supplementary Material online). The sequences with BLAST hit results were further annotated based on human orthologs using GOrilla (Eden et al. 2009) to retrieve functional information from the Gene Ontology (GO) terms under the categories of biological processes, molecular function, and cellular component. We compared the GO terms associated with the genes differentially expressed in the proboscis (target list) with those associated with the rest of the transcriptome (background list) to identify and visualize biological, molecular, and cellular functions that are enriched in the proboscis tissue. The number of genes, *P*-value, and enrichment values associated with each GO term (supplementary file S1 and table S3, Supplementary Material online) were extracted and used to create a bubble graph in R. The sequences without BLAST hit that were found upregulated in the proboscis tissue were further annotated with InterProScan (Jones et al. 2014) to identify known protein domains and motifs.

### Identification of Toxin Orthologs Across Nemertea

To evaluate whether the putative toxins were lineage-specific or found across Nemertea, orthology inferences were carried out using publicly available transcriptomes (Andrade et al. 2014; Halanych and Kocot 2014; Romiguier et al. 2014; Struck et al. 2014; Ament-Velásquez et al. 2016; Plese et al. 2019; Rousselle et al. 2020). List of public transcriptomes used, and corresponding accession numbers can be found in (supplementary file S1 and table S1, Supplementary Material online). Reads from these transcriptomes were downloaded from the SRA database, assembled using Trinity (v. 2.8.4; Grabherr et al. 2011), and the corresponding proteome predicted using TransDecoder v 5.5.0 (Haas et al. 2013). The OrthoFinder v2.3.3 (Emms and Kelly 2015) pipeline was then used to cluster the predicted proteomes from previously published transcriptomes and the set of putative toxins identified here into groups of homologous sequences (orthogroups).

### Proteomic Analyses

To identify peptides and proteins present in the toxic defensive and predatory secretions of *A. valida*, we analyzed a total of five samples: a flash-frozen complete specimen, lyophilized defensive mucus from three individuals, two flash-frozen proboscides, and a laser microdissected proboscis from a KINFix-fixed paraffin-embedded specimen (Stefanits et al. 2016). Lyophilized mucus was dissolved in 300 μl of 30% acetonitrile (ACN) in water and 0.1% formic acid (FA). Whole animal and one flash-frozen proboscis were analyzed using SDS-PAGE followed by in-gel digestion and liquid chromatography tandem mass spectrometry (LC–MS/MS), whereas another flash-frozen proboscis and a laser microdissected proboscis were analyzed by shotgun LC–MS/MS. For SDS-PAGE/in-gel digestion/LC–MS/MS, samples were dried down and the pellets redissolved in (100 μl) of 1D electrophoresis buffer and analyzed by SDS-PAGE. The protein extracts were suspended in a volume up to 50 μl of sample buffer, and then applied onto 1.2-cm wide wells of a conventional SDS-PAGE gel (0.75 mm-thick, 4% stacking, and 10% resolving). The run was stopped as soon as the front entered 3 mm into the resolving gel, so that the whole proteome became concentrated in the stacking/resolving gel interface. The protein bands were visualized by Coomassie staining, excised, cut into cubes (2 × 2 mm), and placed in 0.5 ml microcentrifuge tubes (Moreno et al. 2014). The gel pieces were destained in acetonitrile:water (ACN:H_2_O, 1:1), were reduced and alkylated (disulfide bonds from cysteinyl residues were reduced with 10 mM DTT for 1 h at 56 °C, and then thiol groups were alkylated with 10 mM iodoacetamide for 1 h at room temperature in darkness) and digested in situ with sequencing grade trypsin (Promega, Madison, WI, USA) or chymotrypsin (Roche) as described by Shevchenko et al. (1996) with minor modifications. The gel pieces were shrunk by removing all liquid using sufficient ACN. Acetonitrile was pipetted out and the gel pieces were dried in a speedvac. The dried gel pieces were reswollen in 100 mM Tris–HCl pH 8, 10 mM CaCl_2_ with 60 ng/μl trypsin or chymotrypsin at 5:1 protein:enzyme (w/w) ratio. The tubes were kept in ice for 2 h and incubated at 37 °C (trypsin) or 25 °C (chymotrypsin) for 12 h. Digestion was stopped by the addition of 1% TFA. Whole supernatants were dried down and then desalted onto OMIX Pipette tips C18 (Agilent Technologies) until the mass spectrometric analysis.

The desalted protein digest was dried, resuspended in 10 l of 0.1% FA and analyzed by RP-LC–MS/MS in an Easy-nLC II system coupled to an ion trap LTQ-Orbitrap-Velos-Pro hybrid mass spectrometer (Thermo Scientific). The peptides were concentrated (on-line) by reverse phase chromatography using a 0.1 mm × 20 mm C18 RP precolumn (Thermo Scientific), and then separated using a 0.075 mm × 250 mm C18 RP column (Thermo Scientific) operating at 0.3 μl/min. Peptides were eluted using a 180-min dual gradient. The gradient profile was set as follows: 5−25% solvent B for 135 min, 25−40% solvent B for 45 min, 40−100% solvent B for 2 min, and 100% solvent B for 18 min (solvent A: 0.1% FA in water, solvent B: 0.1% FA, 80% acetonitrile in water). ESI ionization was done using a Nano-bore emitters Stainless Steel ID 30 μm (Proxeon) interface. The Orbitrap resolution was set at 30,000. Peptides were detected in survey scans from 400 to 1,600 amu (1 μscan), followed by 20 data-dependent MS/MS scans (Top 20), using an isolation width of 2 u (in mass-to-charge ratio units), normalized collision energy of 35%, and dynamic exclusion applied during 60 s periods.

Peptide identification from raw data was carried out using PEAKS Studio X search engine (Bioinformatics Solutions Inc.). The predicted proteome (i.e., translated reference transcriptome) was used as a decoy-fusion database to search the resulting MS/MS spectra. The following constraints were used for the searches: tryptic or chymotryptic cleavage (semispecific), up to two missed cleavage sites, and tolerances of 20 ppm for precursor ions and 0.6 Da for MS/MS fragment ions and the searches were performed allowing optional Met oxidation and Cys carbamidomethylation. FDR for peptide spectrum matches was limited to 0.01. Only those proteins with at least two distinct peptides being discovered from LC/MS/MS analyses were considered reliably identified (Han et al. 2011; Zhang et al. 2012).

For shotgun LC–MS/MS analysis, a single proboscis was placed in 100 µl 10% ACN and gently squeezed using a plastic pestle to facilitate the release of soluble toxins. The tissue was removed, the liquid centrifuged at maximum speed (>18k rcf), the protein concentration was estimated by measuring the absorbance at 280 nm using a NanoDrop 2000 (Thermo Scientific). The supernatant containing 5 µg protein was made to a final concentration of 50 mM ammonium bicarbonate 10% ACN 2 M urea in 8 µL, reduced and alkylated as above, and digested with 30 ng/µl trypsin at 37 °C overnight. The resulting tryptic peptides were desalted using a C18 ZipTip (Thermo Scientific, USA), dried using vacuum centrifugation, dissolved in 0.5% FA, and 2 μg analyzed by LC–MS/MS on a Sciex 5600 TripleTOF equipped with a Turbo-V source heated to 550 °C. The dissolved samples were fractionated on a Shimadzu (Kyoto, Japan) Nexera UHPLC with an Agilent Zorbax stable-bond C18 column (Agilent, USA) (2.1 × 100 mm, 1.8 μm particle size, 300 Å pore size), using a flow rate of 180 μl/min and a gradient of 1–40% solvent B (90% ACN, 0.1% FA) in 0.1% FA over 60 min.

MS1 spectra were acquired at 300–1,800 m/z with an accumulation time of 250 ms, and selecting the 20 most intense ions for MS2. Precursor ions with a charge of +2 to +5 and an intensity of at least 120 counts/s were selected, with a unit mass precursor ion inclusion window of 0.7 Da, and isotopes within 2 Da were excluded. MS2 scans were acquired at 80–1,400 m/z, with an accumulation time of 100 ms, and optimized for high resolution. The resulting MS/MS spectra were then searched against the translated reference transcriptome with added common contaminants using Protein Pilot v5.0 (AB SCIEX, USA), allowing for biological modifications and amino acid substitutions in order to account for potential between-specimen toxin variance. False positives were identified using decoy-based FDR as estimated by Protein Pilot, and only protein identifications with a corresponding local FDR of <0.5% were considered significant. The mass spectrometry proteomics data have been deposited to the ProteomeXchange Consortium via the PRIDE (Perez-Riverol et al. 2022) partner repository with the dataset identifier PXD033380.

### MALDI Imaging Mass Spectrometry

To visualize the distribution of peptides in the proboscis of *A. valida*, the tissue was processed and embedded in paraffin using the same protocol as described previously (Undheim et al. 2014; Mitchell et al. 2017), which removes lipids as well as most nonfixed, nonpeptidic analytes, and thereby improves signals from peptidic analytes. Sections were cut at 7 µm thickness from cold paraffin block using a microtome, placed directly onto an indium-tin oxide glass slide to which they were attached by heating to 57 °C. The sections were then allowed to cool before the paraffin was removed using xylene and matrix (105 mg α-cyano-4-hydroxycinnamic acid [CHCA], 8 ml acetonitrile, 7 ml water, 30 µl trifluoroacetic acid) was applied with an ImagePrep vibrational vaporization-deposition system (Bruker) using the standard Bruker ImagePrep CHCA application method. MSI data were acquired using Flex Imaging 4.1 and Flex Control 3.4 to operate a MALDI-TOF/TOF (Bruker Ultraflex III, Bruker, Bremen, Germany) in linear positive mode. Scans were acquired over a mass range of m/z 1,000–15,000 at 200 Hz, 50 µm spatial resolution, 600 shots, and using a medium laser size. Data were analyzed postacquisition using Flex Imaging 4.1 and SCILs LAB.

## Results and Discussion

### Gene Expression and Functional Enrichment in the Hoplonemertean Proboscis

We used comparative transcriptomics and RNA-seq DGE analysis to investigate the molecular toolkit upregulated in the proboscis organ. We sequenced a total of 12 RNA libraries corresponding to the proboscis and the body of six specimens of *A. valida*. We merged the reads of the 12 replicated libraries to build a reference transcriptome generating a total of 681,621 transcripts. Raw sequencing data, assembly statistics, and completeness metrics can be found in (supplementary file S1 and table S2, Supplementary Material online). After mapping the individual libraries of each replicate to the reference transcriptome and performing the DGE analysis, we identified 4,713 genes that showed differential expression between the proboscis and the posterior end. A total of 1,337 genes were upregulated in the proboscis, whereas 3,375 were upregulated in the posterior end of the body tissue (supplementary file S3, Supplementary Material online). Of these differentially expressed genes, we could only annotate 327 (ca. 24%) of the ones upregulated in the proboscis and 1,421 (ca. 42%) of those upregulated in the body. We focused our downstream analyses on the annotated genes upregulated in the proboscis because it is a unique structure in the phylum Nemertea, specifically used for prey capture.

This unique structure, the ribbon worm proboscis, is a long eversible muscular organ with tubular shape formed by an invagination of the anterior end of the body (Gibson 1972; Mcdermott and Roe 1985). The central lumen of the proboscis is lined by a glandular epithelium that becomes the outer layer and it is therefore exposed to the external environment when the organ is everted. The various types of gland cells present in this epithelium produce a sticky, toxic mucus secretion that might increase adhesion of the proboscis to the prey and/or substratum and cause paralysis and/or death of the captured organisms (Stricker and Cloney 1983; Kem 1988; Montalvo et al. 1997; Montalvo et al. 1998; Jennings and Gibson 2016). Although the proboscis has been mainly associated with prey capture, other functions have also been suggested including defense, locomotion, burrowing, adhesion, and sensory probing (e.g., Gibson 1972; Stricker and Cloney 1981; Stricker and Cloney 1983; Mcdermott and Roe 1985; Junoy et al. 2001; Magarlamov et al. 2021). However, these functions have been mostly inferred from behavioral or microscopical observations and to our knowledge, there are no molecular studies supporting the role of the proboscis in these additional functions.

**Fig. 2.**
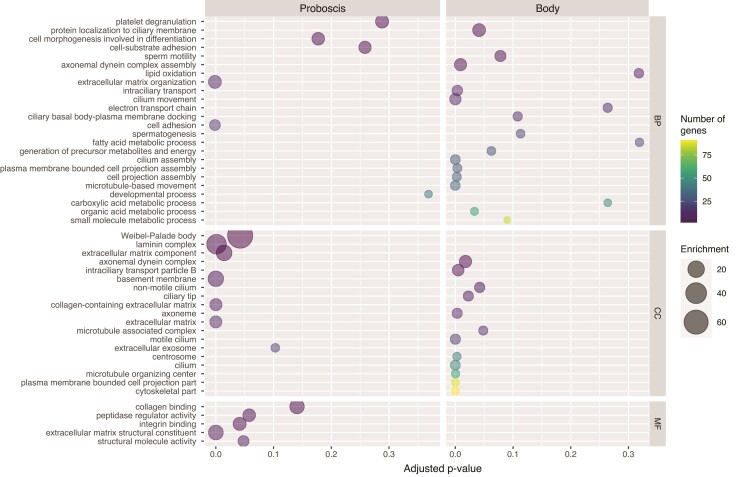
Bubble plot showing functional enrichment in the proboscis and body tissue of *Antarctonemertes valida*. The bubbles indicate enriched GO terms in the biological process (BP), cellular compartment (CC), and molecular function (MF) categories extracted from the most upregulated genes in the two analyzed tissues.

In our analyses, we could retrieve functional information from 327 genes of those found upregulated in the proboscis, which allowed us to identify various enriched GO terms relevant to the physiology of this predatory structure. Most up-regulated genes were involved in functions related to secretion, immune response, cell adhesion, and the nervous and neuromuscular systems (fig. 2, supplementary file S1 and table S3, Supplementary Material online). Remarkably, the great majority of the GO terms found enriched in the proboscis, including cell morphogenesis involved in differentiation, cell-substrate adhesion, extracellular matrix organization, extracellular exosome, collagen binding, or structural molecule activity (fig. 2) have been also found enriched in a recent comparative analyses of venom gland transcriptomes across the Metazoa (Zancolli et al. 2022). Some of these enriched functions are related to tissue development and cell cycle regulation and are indicative of high epithelial cell turnover, which would be necessary to repair or replace damaged secreting cells after toxin release (Zancolli et al. 2022). This suggests that the nemertean proboscis, despite not being a distinct multicellular venom gland, has similar global gene expression patterns as those of animals such as snakes, scorpions, and spiders.

**Fig. 3.**
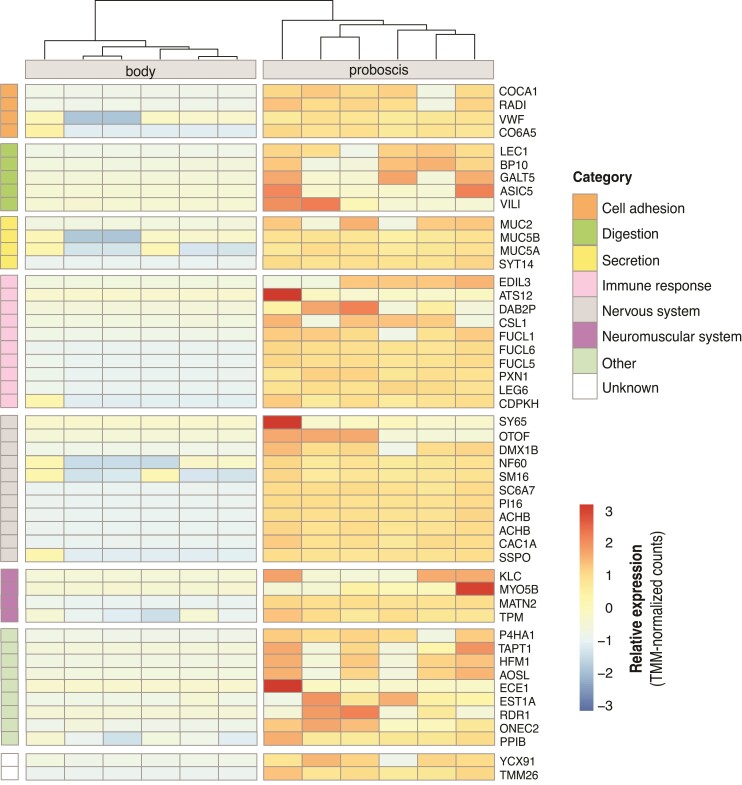
Hierarchically clustered heatmap showing expression levels of the 50 most upregulated genes in the proboscis samples. Genes are grouped in eight general categories according to their associated functions. Relative expression levels (TMM-normalized counts) are indicated by a color gradient from blue (low) to red (high).

In addition, some of these enriched functions, such as those related to secretion (Weibel-Palade body and peptidase regulator activity) and cell adhesion (basement membrane, laminin complex, and collagen-containing extracellular matrix) provide evidence supporting the suggested role of the proboscis secretions in increasing adhesion to the prey and/or substratum (Gibson 1972; Stricker and Cloney 1981; Mcdermott and Roe 1985). Interestingly, enrichment of platelet degranulation could point to a mechanism of induced inflammation and blood coagulation by platelet-activating toxins similar to that of viperid snakes (Teixeira et al. 2019). In fact, we found several upregulated genes in the proboscis tissue that are involved in inflammation, innate immune response, and host defense against microorganisms. For example, we found homologs of *pentraxin fusion protein* (*PXN1*) which functions as a pattern recognition molecule against foreign antigens (du Clos 2013), *galectin-6* (*LEG6*) which is a skin antimicrobial peptide (Natsuga and Watt 2016), and several *fucolectins* (*FUCL1*, *FUCL5*, *FUCL6*), which act as defensive agents in the innate immune system (Honda et al. 2000) (fig. 3, supplementary file S3, Supplementary Material online).

The majority of genes found upregulated in the proboscis of *A. valida* are involved in functions related to cell adhesion, secretion, immune response, and to the nervous and neuromuscular systems (fig. 3, supplementary file S3, Supplementary Material online). For instance, we found several upregulated genes involved in cell adhesion, motility, and proliferation including *collagen alpha-1(XII) chain* (*COCA1*), *radixin* (*RADI*), and *von Willebrand factor* (*VWF*) (Dejana et al. 1989; Hoeflich and Ikura 2004). We also found *polypeptide N-acetylgalactosaminyltransferase 5* (*GALT5*), an ortholog of *GALNT12* expressed mainly in the stomach, small intestine, and colon that may play an important role in the initial step of mucin-type oligosaccharide biosynthesis in digestive organs (Brown et al. 2021), as well as several *mucins* (*MUC2*, *MUC5A*, *MUC5B*), glycoproteins that coat gastric and respiratory tract epithelia providing protection and lubrication against infections and chemical damage (Coleman and Haller 2018; Brown et al. 2021). The inherent secretory nature of the proboscis is supported by the presence of upregulated genes generally involved in exocytosis and secretion such as *anoctamin-7* (*ANO7*) and *synaptotagmin-14* (*SYT14*). Anoctamins specifically, are Ca^2+^-activated Cl^−^ channels that are involved in fluid secretion in a variety of secretory epithelial cells coating airways, intestines, and salivary glands (Hartzell et al. 2009; Jang and Oh 2014). Interestingly, *anoctamin* is also expressed in zebrafish skeletal muscle, where it plays an essential role in action potential acceleration, resulting in controlled, faster, and stronger muscle contractions which are crucial for high-speed movement (Dayal et al. 2019). These genes could therefore have a role in the secretion of toxins and other proteins in the toxic mucus produced by the various types of gland cells that form the proboscis epithelium, but also might be involved in accelerating the action potential of muscle cells, allowing ribbon worms to use extremely fast proboscis strikes for hunting.

The high-speed movements and maintenance of force by the proboscis musculature when gripping the prey might be facilitated by other genes that were also found upregulated in the proboscis, including *60 kDa neurofilament protein* (*NF60*), *titin* (*TITIN*), and *twitchin* (*UNC22*). *Neurofilaments* (*NFs*) are the major component of neuron axonal intermediate filaments and are required for the development of normal axonal caliber, a property that determines the velocity of axonal conduction (Szaro et al. 1991). In invertebrate axons in particular, the speed of conduction of an action potential is proportional to the axon diameter and thus, invertebrates have large-diameter axons for signals that need to propagate rapidly. The squid giant axon for example, which is involved in rapid propulsion during the escape response, can reach a diameter of up to 1 mm (Caldwell 2009). We found homologs of squid giant axon NFs upregulated in the proboscis, which suggests that ribbon worms might also have giant axons in the proboscis, an organ that necessitates rapid responses for predation or defense. The upregulated *titin* and *twitchin* homologs, key components in the assembly and functioning of striated muscle, and regulators of muscle contraction and relaxation respectively, might be involved in the elasticity and force maintenance of the proboscis. *Titin* is a giant protein that functions as a molecular spring and is responsible for the passive elasticity of muscle (Siegman et al. 1998), whereas *twitchin* has been shown to regulate catch muscle contraction, a unique phenomenon of prolonged, high-force maintenance with very low energy consumption that occurs in some invertebrate smooth muscles, including the byssus retractor muscle of the mussel *Mytilus edulis* (Shelud’ko et al. 2004; Funabara et al. 2005).

We also found several upregulated genes in the proboscis tissue that are related to sensory perception, which would support hypotheses and observations by previous authors related to the use of the proboscis as an exploratory organ for sensory probing (Gibson 1972; Magarlamov et al. 2021). Some of these genes include *acid-sensing ion channels* (*ASIC5*, *ASI1B*) which sense reduced levels of extracellular pH resulting in a neuronal signal. They are typically found in skin, muscles, and viscera nerve fibers where they are associated with pain, taste, and gastrointestinal functions (Sluka et al. 2009). We also identified upregulated *cadherin-23* (*CAD23*) homologs which are calcium-dependent cellular adhesion proteins that interlink epithelial sensory cells. Specifically, *cadherin-23* connects apical bundles of stereocilia on hair cells and is essential for inner-ear mechanotransduction, initiating sensory perception in response to mechanical stimuli (Jaiganesh et al. 2018).

Interestingly, although we found several putative novel predatory and defensive toxins (see following section), we did not find any homologs of the few previously known heteronemertean toxins (e.g., parborlysin, cytotoxin A-III, neurotoxin B-IV, α- and β-nemertides). This is in line with previous studies that have also failed to identify heteronemertean toxins in hoplonemerteans and vice versa, suggesting that different lineages of ribbon worms have distinct toxin cocktails (see also section “Lineage-specificity of novel defensive and predatory toxins across Nemertea”). In addition, whereas we did identify a few homologs of the recently described U-nemertotoxins from the hoplonemertean *A. lactifloreus* (Johnston 1828; von Reumont et al. 2020), and homologs of additional toxins from other venomous organisms, these were not differentially expressed in the proboscis. This might suggest these toxins are used both in predation and defense since their expression levels are comparable between the proboscis and body wall, or alternatively that they are nontoxin homologs expressed throughout the body to fulfill a different function.

### Proteotranscriptomic Characterization of Putative Predatory and Defensive Toxins

Identifying toxins and other venom components in nemerteans, especially those secreted by the proboscis, is challenging due to the lack of a multicellular venom gland and the impossibility of milking the venom to analyze the isolated secretion. Therefore, to identify toxin candidates in the hoplonemertean *A. valida*, whereas minimizing false positives and annotation error rates, we followed a proteotranscriptomic approach, combined with different lines of evidence including DGE analyses and MALDI-IMS (Smith and Undheim 2018). We identified a total of 4,539 predicted proteins that were present in at least one of the five proteomes analyzed. Among these, we identified 85 putative hoplonemertean toxins that we further classified into potential predatory, defensive, and dual-function toxins based on their expression patterns and proteomic distribution (table 1, supplementary file S4, Supplementary Material online). Specifically, we identified (1) 26 putative predatory toxins that were proboscis-specific, that is differentially upregulated in the proboscis, present in at least one proboscis proteome and absent in the mucus proteome; (2) 14 putative defensive toxins that were body-specific, that is differentially upregulated in the body wall tissue, present in the mucus proteome, and absent from all the proboscis proteomes; (3) six putative toxins of dual defensive and predatory function that were present in both mucus and proboscis proteomes; and finally, (4) 39 putative toxins that we could not clearly classify into any of the above categories (supplementary file S4, Supplementary Material online).

**Table 1.**
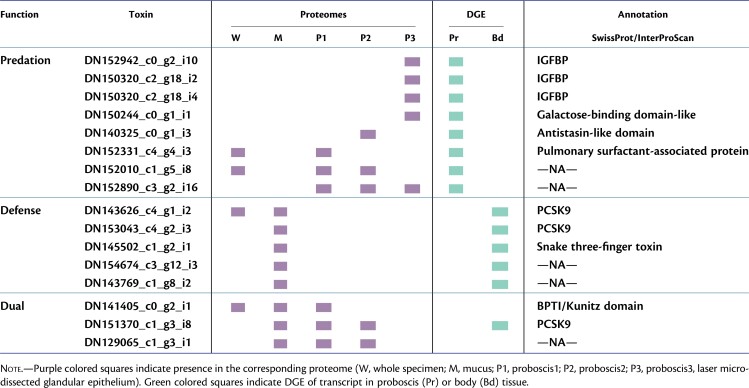
Selected List of Putative Predatory, Defensive, and Dual-Function Toxins Identified in *A. valida.*

**Fig. 4.**
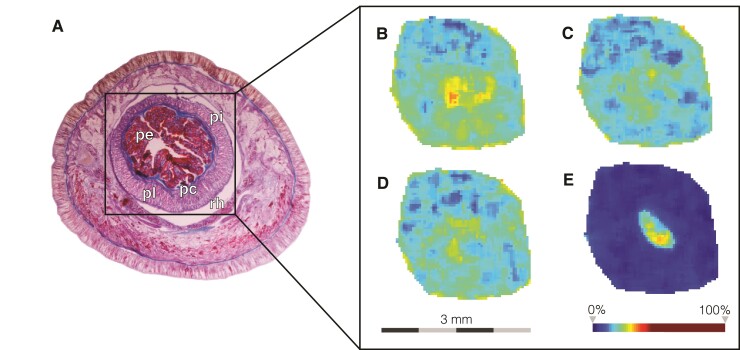
MALDI-IMS data for the proboscis of *Antarctonemertes valida*. (*A*) Histological image of an HE-stained transversal section of the anterior end of an *A. valida* specimen. (*B–E*) Spatial distribution pattern of putative peptides showing higher intensity (yellow/orange) in the central glandular epithelium. (*B*) Peptide observed at 4,420.19 m/z corresponding to putative predatory toxin containing IGFBP (DN150320_c2_g18_i4); (*C*) peptide observed at 3,355.83 m/z corresponding to putative predatory toxin containing galactose-binding-like domain (DN150244_c0_g1_i1); (*D*) putative peptide observed at 3,543.12 m/z corresponding to putative predatory toxin antistasin-like domains (DN140325_c0_g1_i3); (*E*) putative peptide observed at 5,324 m/z. pe, proboscis epithelium; pi, proboscis inner lining layer; pc, proboscis circular muscle layer; pl, proboscis longitudinal muscle layer; rh, rhynchocoel.

Among the putative predatory toxins, we identified several transcripts with insulin-like growth factor binding protein (IGFBP) domains (table 1, supplementary file S4, Supplementary Material online). IGFBP-like proteins have been found in other animal venoms, including scorpions, spiders, and cnidarians, and more recently in the hoplonemertean *A. lactifloreus* (Ward, Ellsworth, and Rokyta 2018; Paiva et al. 2019; Klompen et al. 2020; von Reumont et al. 2020). Their specific function in the venom cocktail is not clear; they may act as venom-spreading factors, although there is also evidence of their potential toxic effect (Paiva et al. 2019; von Reumont et al. 2020). Other transcripts identified here as putative predatory toxins have galactose-binding-like domains (table 1, supplementary file S4, Supplementary Material online). Proteins containing this domain fold can be found in different families, including some often present in animal venoms such as snake venom galactose-binding lectins (Sartim and Sampaio 2015); nattectins, which are C-type lectins from the venomous fish *Thalassophryne nattereri* that have been also recently identified in the proteotranscriptome of the hoplonemertean *A. lactifloreus* (Lopes-Ferreira et al. 2011; von Reumont et al. 2020); and SUL-I galactose-binding lectins present in the venom of the flower sea urchin *Toxopneustes pileolus* (Hatakeyama et al. 2017). These proteins have been reported to mediate several biological functions associated with envenomation including platelet aggregation and inflammation, as well as mitogenic, chemotactic, and cytotoxic activities (Edo et al. 2012; Sartim and Sampaio 2015). We also identified putative predatory toxins with antistasin-like domains (table 1, supplementary file S4, Supplementary Material online), which are common anticoagulants in leech saliva and have been also recorded from other nonblood-feeding invertebrates such as cnidarians and mollusks where they may act as modulators of the immune response (Iwama et al. 2021). To provide more evidence about the toxic nature and potential predatory role of these putative toxins, we looked at their spatial distribution within the *A. valida* proboscis tissue by MALDI-IMS. The putative predatory toxins discussed above all seem to be mainly localized to the central core of the inverted proboscis (fig. 4, supplementary file S5 and figs. S2–S7, Supplementary Material online), which corresponds to the glandular epithelium that secretes the sticky toxic mucus used for prey capture and becomes exposed to the external environment when the organ is everted for hunting. We also identified additional peptides with intense signals, whose distributions were clearly restricted to the glandular epithelium of the proboscis but that we could not match to any of the transcripts identified here (fig. 4, supplementary file S5 and fig. S12, Supplementary Material online). These might represent novel predatory toxins that were not being expressed in the individuals used for RNA sequencing and should be further investigated in future studies.

Among the putative defensive toxins, we identified some transcripts that belong to the proprotein convertase subtilisin/kexin type 9 (PCSK9) superfamily (table 1, supplementary file S4, Supplementary Material online). PCSK9 is known to mediate cholesterol homeostasis, but it may also participate in systemic immune activation, promoting inflammation, and atherogenesis (Zanni et al. 2017). We also identified putative defensive toxins that belong to the snake three-finger toxin (3FTx) domain superfamily, which is predominantly found in small neurotoxins and cytotoxins from snake venoms that bind to nicotinic acetylcholine receptors (nAChRs) at neuromuscular junctions causing paralysis (Kini and Doley 2010).

In addition, we identified a handful of putative dual-function toxins that might assist in predation as well as in defense as they were found in both mucus and proboscis proteomes (table 1, supplementary file S4, Supplementary Material online), including a homolog of Kunitz-type U19-barytoxin-TI1a from the spider *Trittame loki*. Kunitz-type toxins have been isolated from a variety of venomous and blood-feeding animals such as snakes, bees, ticks, sea anemones, scorpions, and spiders and interfere with different physiological processes such as blood coagulation, inflammation, or blocking ion channels (Calvete et al. 2007; Choo et al. 2012; Isaeva et al. 2012; Soares et al. 2012; Wan et al. 2013; Ding et al. 2015).

Lastly, we identified 39 additional putative toxins that we could not clearly assign to any of the functional classes discussed above (i.e., predatory, defensive, and dual-function toxins) (supplementary file S4, Supplementary Material online). These include several actitoxin-like and plancitoxin-like peptides, homologous to the recently proposed nemertean toxin classes U-nemertotoxin-1 and U-nemertotoxin-2 which might have a neurotoxic effect aiding in the paralysis of the prey (von Reumont et al. 2020). Other putative toxins in this group have ShK domains, potent inhibitors of potassium channels originally characterized in sea anemones (Castañeda et al. 1995) and later identified in a variety of other venomous organisms including jellyfish, ribbon worms, and vampire snails (Whelan et al. 2014; Modica et al. 2015; Ponce et al. 2016). Most cnidarian ShK toxins cause paralysis, although some show haemolytic effects and others might also act as anesthetics (Modica et al. 2015). Interestingly, we also found putative toxins that show homology to the neuropeptide vasotocin–neurophysin (Cruz et al. 1987; Nielsen et al. 1994; Dutertre et al. 2008) contributing to the growing evidence that hormone-like peptides targeting neuroendocrine processes might be an important component of animal venoms (Safavi-Hemami et al. 2015; Undheim, Grimm, 2015; Undheim, Hamilton, 2015; Robinson et al. 2017; Sachkova et al. 2020). Nevertheless, a more in-depth evaluation of the molecular architecture, domain organization, and sequence/structure conservation of the putative toxins identified here is necessary to elucidate their specific functions.

It is also worth noting that the annotation of our reference transcriptome by BLAST recovered a total of 70 transcripts that showed homology with known toxins from other animal venoms (supplementary file S2, Supplementary Material online), including several neurotoxins such as the potassium channel pore-blocking conkunitzin (Bayrhuber et al. 2005) and the crustacean-specific alpha-latrocrustatoxin (Rohou et al. 2007) and enzymes like astacin-like metalloprotease that might play a role in extraoral digestion in spiders (Walter et al. 2017). Although these transcripts are interesting, only 12 of them were present in the proteomes analyzed and therefore those are the only ones we considered putative toxins and further discussed above. This further highlights the importance of including several lines of evidence when investigating the toxic secretions of understudied venomous organisms (Smith and Undheim 2018).

### Lineage-Specificity of Novel Defensive and Predatory Toxins Across Nemertea

**Fig. 5.**
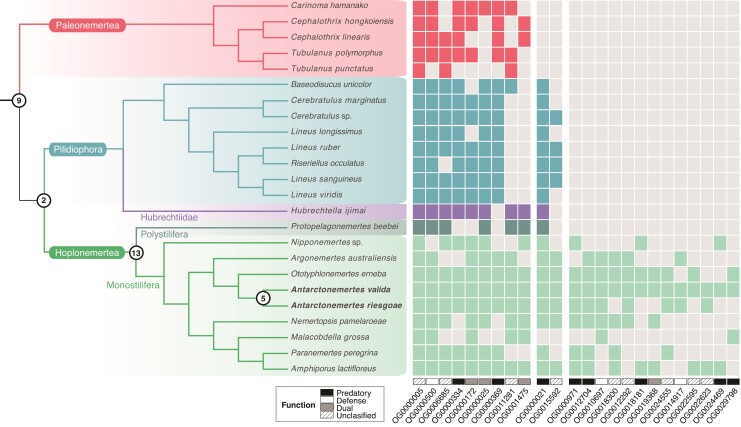
Phylogeny of Nemertea based on Andrade et al. (2014) indicating presence (colored squares) or absence (gray squares) of each of 24 toxin orthogroups in the species analyzed. The remaining six toxin orthogroups that include only *Antarctonemertes* sequences are not shown. Numbers over nodes indicate the shared orthogroups for the corresponding clade. Orthogroup ID and putative function of the corresponding toxins in each orthogroup are denoted at the bottom.

Parallel evolution of venom components is pervasive among venomous animals (Jenner et al. 2019; Barua et al. 2021; Zancolli et al. 2022) but given the dearth of data related to venom composition in the Nemertea, this has never been assessed before in the phylum. Here, to investigate the evolution of the 85 putative toxins identified through proteotranscriptomics, we used OrthoFinder to search for their orthologues in publicly available nemertean transcriptomes. Our results show that 56 of these putative toxins clustered in 30 different orthogroups with sequences from other nemertean species (fig. 5), whereas the 29 remaining putative toxins seem to be unique for *A. valida*, with no orthologs found in any of the analyzed transcriptomes. Of the 30 shared orthologous toxins clusters, 9 are common to all analyzed ribbon worm species including representatives of Palaeonemertea, Pilidiophora, and Hoplonemertea, 2 are shared by Pilidiophora and Hoplonemertea, 13 seem to be lineage-specific as they exclusively include sequences from hoplonemertean species (fig. 5), and 6 are further restricted to the genus *Antarctonemertes* (not shown in fig. 5). These results suggest that the evolution of toxic secretions is highly divergent across lineages, producing toxin cocktails that largely vary across nemerteans with a high proportion of lineage-specific toxins that might be finely tuned to the predatory habits of their members. In fact, variation in toxin composition has been reported in a variety of venomous taxa as a result of diverse factors including sex (Ward, Ellsworth, Hogan, et al. 2018), ontogeny (Cipriani et al. 2017), and diet (Phuong et al. 2016; Cipriani et al. 2017). Specifically, evidence for diet-related selection pressures associated with predator–prey interactions is ample, with numerous studies showing that the venom of trophic specialists is more toxic to its preferred prey than to alternative prey (Richards et al. 2012; Pekár, Petráková, et al. 2018; Pekár, Líznarová, et al. 2018). Nemerteans are highly selective predators, with heteronemerteans primarily feeding on polychaete annelids and hoplonemerteans on crustaceans, especially amphipods (Gibson 1972; Mcdermott and Roe 1985; Thiel and Kruse 2001). In fact, evidence from feeding experiments shows that organisms that are not normally part of the diet of a particular nemertean species are only slightly or not at all affected by its proboscis secretions (Gibson 1972; ROE 1976), suggesting dietary specialization might be an important driver of venom composition in ribbon worms. *Antarctonemertes valida* and its congeneric *A. riesgoae* (Taboada et al. 2013) are relatively common in shallow waters of the Southern Ocean (Taboada et al. 2013; Taboada et al. 2018) and although no direct evidence exists on their trophic preferences, the amphipod *Cheirimedon femoratus* (Pfeffer 1888) might represent one of their main food sources as it reaches remarkable densities in the same habitats (e.g., Aghmich et al. 2016; Angulo-Preckler et al. 2017).

Since lineage-specificity of certain toxins and venom components is often related to prey preferences, dietary shifts may render certain toxins useless, and eventually the animals might lose the corresponding coding genes. For example, Dowell et al. (2016) found that a *phospholipase A2* gene complex encoding a family of venom toxins expanded early in rattlesnake evolution, and more recently, neurotoxin or myotoxin genes were independently deleted in different lineages reflecting shifts in prey choice that made some toxins dispensable (Dowell et al. 2016). Conversely, it has also been proposed that the massive lineage-specific expansion and diversification of *SVMP* genes in viperids and crotalids is a result of positive selection, comparable to the diversification of antifreeze glycoprotein genes in Antarctic fish as an adaptation to specific environmental conditions (Giorgianni et al. 2020). It has also been shown that novel lineage-specific genes in cnidarians, particularly those related to venom and venom delivery, have undergone increased expansion events compared with gene families with wider taxonomic distributions, and evolve through lineage-specific duplications and divergent selective pressures (Surm et al. 2019). In scorpions, calcins and LKTx toxins are phylogenetically restricted to the Iurida and Buthida lineages, respectively, and analysis of sequences and molecular models show such a remarkable phylogenetic inertia that the morphology of each of these toxins has been proposed as a synapomorphy for the respective clades (Santibáñez-López et al. 2018). This taxonomically restricted evolution is also common in centipede venom, as evidenced by numerous lineage-specific toxin families comprising stinkingly unique complex venoms that have diverged substantially from a simpler ancestral venom through gene duplications, and frequent functional recruitments and losses of toxin gene families (Jenner et al. 2019).

Whether nemertean venoms evolved through recurrent functional recruitments and losses of toxin gene families, or through lineage-specific expansions and functional diversification events as a result of dietary specializations or alternative selective pressures remains unclear and will have to be investigated in future studies including a wider taxonomical coverage. However, our results indicate that toxin composition of nemertean venoms is highly divergent across lineages, producing distinct toxin cocktails with a high proportion of lineage-specific toxins that shape diverse ribbon worm venom arsenals across the phylum.

## Conclusion

Ribbon worms are active predators that use an eversible proboscis to inject venom into their prey and defend themselves with toxic epidermal secretions. Previous work has focused on just a handful of species, identifying a few nonproteinaceous toxins, cytolytic, and neurotoxic peptides (Kem 1976; Ali et al. 1990), whereas a couple of more recent studies using high-throughput technologies discovered a much greater diversity of toxin-like genes than were previously documented (Whelan et al. 2014; von Reumont et al. 2020), suggesting there is a significant undiscovered diversity of ribbon worm toxins. In fact, these neglected venomous invertebrates have recently received increased attention as sources of novel bioactive peptides with translational potential into eco-friendly pesticides (Jacobsson et al. 2018); however, our understanding of the composition and evolution of ribbon worm venoms is still very limited.

To our knowledge, this is the first comparative study of ribbon worm venom that combines gene expression profiling and MSI of venom-producing tissues with proteotranscriptomics of toxic secretions to shed light onto the composition and evolution of nemertean predatory and defensive venoms. Our results show that ribbon worms produce distinct toxin cocktails for predation and defense with numerous lineage-specific toxins, suggesting that venom evolution is highly divergent across nemerteans, producing venoms that might be finely tuned to subdue different prey. Our results advance our knowledge into the diversity and evolution of animal venoms and highlight the importance of combining different data to characterize toxins in poorly known venomous organisms.

## Supplementary Material

Supplementary data are available at *Molecular Biology and Evolution* online.

## Supplementary Material

msac096_Supplementary_Data

## Data Availability

The data underlying this article are available in NCBI SRA database at https://www.ncbi.nlm.nih.gov/sra and can be accessed with accession numbers listed in supplementary file 1, table 1, Supplementary Material online; and in ProteomeXchange Consortium via the PRIDE partner repository at http://proteomecentral.proteomexchange.org/cgi/GetDataset and can be accessed with the dataset identifier PXD033380.
