# Supplementary material for: Evolution, Expression Patterns, and Distribution of Novel Ribbon Worm Predatory and Defensive Toxins
Source: Mol Biol Evol. 2022 May 5;39(5):msac096. doi: 10.1093/molbev/msac096 (PMC9132205; doi:10.1093/molbev/msac096)
Supplement: msac096_Supplementary_Data [file msac096_supplementary_data.zip › Supplementary_file1.docx]

Supplementary Material online.

Verdes et al 2022. Expression patterns and distribution of novel predatory and defensive toxins in the hoplonemertean Antarctonemertes valida (Nemertea).

**Supplementary Table 1**. List of accession numbers for the NCBI-SRA repository bioprojects where the datasets generated and analyzed in this work have been deposited.

| **Species** | **NCBI-SRA** | **Reference** |
| --- | --- | --- |
| *Antarctonemertes valida* | PRJNA831820 | This study |
| *Antarctonemertes riesgoae* | PRJNA831820 | This study |
| *Argonemertes australiensis* | PRJNA254358 | Andrade *et al*. (2014) |
| *Baseodiscus unicolor* | PRJNA322119 | Andrade *et al*. (2014) |
| *Carinoma hamanako* | PRJNA254071 | Andrade *et al*. (2014) |
| *Cephalothrix hongkongiensis* | PRJNA181263 | Andrade *et al*. (2014) |
| *Cephalothrix linearis* | PRJNA245790 | Struck *et al.* 2014 |
| *Cerebratulus marginatus* | PRJNA181261 | Andrade *et al*. (2014) |
| *Cerebratulus sp.* | PRJNA275078 | Egger *et al*. 2015 |
| *Hubrechtella ijimai* | PRJNA254167 | Andrade *et al*. (2014) |
| *Lineus longissimus* | PRJNA330781 | Jacobson *et al.* (2018) |
| *Lineus ruber* | PRJNA249058 | Romiguier *et al.* (2014) |
| *Lineus sanguineus (Argentina)* | PRJNA322119 | Ament-Velazquez *et al*. (2016) |
| *Lineus sanguineus (Brazil)* | To be submitted | -- |
| *Lineus sanguineus (France)* | PRJNA322119 | Ament-Velazquez *et al*. (2016) |
| *Lineus viridis* | PRJNA322119 | Ament-Velazquez *et al*. (2016) |
| *Malacobdella grossa* | PRJNA254359 | Andrade *et al*. (2014) |
| *Nemertopsis pamelaroeae* | To be submitted | -- |
| *Nipponnemertes sp.* | PRJNA254364 | Andrade *et al*. (2014) |
| *Ototyphlonemertes erneba* | To be submitted | -- |
| *Paranemertes peregrina* | PRJNA254365 | Andrade *et al*. (2014) |
| *Protopelagonemertes beebei* | PRJNA254366 | Andrade *et al*. (2014) |
| *Ramphogordius lacteus* | PRJNA530965 | Rousselle *et al*. (2020) |
| *Riseriellus occultus* | PRJNA254175 | Andrade *et al*. (2014) |
| *Tubulanus polymorphus* | PRJNA263418 | Halanych and Kocot (2014) |
| *Tubulanus punctatus* | PRJNA254065 | Andrade *et al*. (2014) |

**Supplementary Table 2**. Sequencing, *de novo* transcriptome assembly metrics and transcriptome completeness statistics based on BUSCO analysis with the Metazoa gene set.

|  | ***Antarctonemertes valida*** | ***Antarctonemertes riesgoae*** |
| --- | --- | --- |
| **Sequencing and assembly statistics** |  |  |
| Raw reads | 407,473,408 | 71,535,679 |
| Trimmed reads | 292,333,312 | 51,832,072 |
| Percent GC | 38.06 | 39.22 |
| Total trinity ‘genes’ | 550,341 | 192,193 |
| Total trinity transcripts | 681,621 | 100,829 |
| Min contig length | 201 | 201 |
| Max contig length | 20,736 | 18,501 |
| Mean contig length | 571 | 541 |
| Median contig length | 357 | 338 |
| Average contig | 570.66 | 591.99 |
| Contig N50 | 696 | 672 |
| Total assembled bases | 388,971,583 | 54,595,127 |
| **Transcriptome Completeness** |  |  |
| BUSCOs Metazoa set | 978 | 978 |
| Complete - single copy | 380 (38.9%) | 624 (63.8%) |
| Complete - duplicated | 568 (58.1%) | 125 (12.8%) |
| Complete total | 948 (96.93%) | 749 (76.58%) |
| Fragmented | 19 (1.9%) | 188 (19.2%) |
| Complete + partial | 967 (98.88%) | 937 (95.81%) |
| Missing BUSCOs | 11 (1.12%) | 41 (4.19%) |

**Supplementary Table 3**. Enrichment analysis showing over-represented Gene Ontology Terms in the proboscis tissue corresponding to Biological Process (BP), Cellular Compartment (CC) and Molecular Function (MF) categories.

| **Category** | **ID** | **Term** | **Adj-pval** | **Enrichment** | **Genes** |
| --- | --- | --- | --- | --- | --- |
| BP | GO:0030198 | extracellular matrix organization | 6.97E-05 | 8.88 | 12 |
| BP | GO:0007155 | cell adhesion | 1.01E-04 | 4.77 | 18 |
| BP | GO:0022610 | biological adhesion | 8.07E-05 | 4.71 | 18 |
| BP | GO:0043062 | extracellular structure organization | 7.34E-05 | 7.84 | 12 |
| BP | GO:0000904 | cell morphogenesis involved in differentiation | 1.79E-01 | 8.24 | 6 |
| BP | GO:0031589 | cell-substrate adhesion | 2.60E-01 | 7.48 | 6 |
| BP | GO:0002576 | platelet degranulation | 2.88E-01 | 9.35 | 5 |
| BP | GO:0032502 | developmental process | 3.70E-01 | 1.62 | 39 |
| BP | GO:0034446 | substrate adhesion-dependent cell spreading | 6.04E-01 | 10.77 | 4 |
| CC | GO:0031012 | extracellular matrix | 1.30E-05 | 6.88 | 14 |
| CC | GO:0005604 | basement membrane | 1.34E-05 | 16.32 | 8 |
| CC | GO:0062023 | collagen-containing extracellular matrix | 1.44E-04 | 7.12 | 11 |
| CC | GO:0043256 | laminin complex | 1.21E-03 | 33.66 | 4 |
| CC | GO:0043259 | laminin-10 complex | 1.01E-03 | 67.31 | 3 |
| CC | GO:0005606 | laminin-1 complex | 8.42E-04 | 67.31 | 3 |
| CC | GO:0044421 | extracellular region part | 7.04E-03 | 2.13 | 28 |
| CC | GO:0044420 | extracellular matrix component | 1.43E-02 | 16.83 | 4 |
| CC | GO:0033093 | Weibel-Palade body | 3.88E-02 | 67.31 | 2 |
| CC | GO:0005615 | extracellular space | 3.57E-02 | 3.09 | 13 |
| CC | GO:0043260 | laminin-11 complex | 9.44E-02 | 44.87 | 2 |
| CC | GO:0070062 | extracellular exosome | 1.03E-01 | 2.1 | 20 |
| CC | GO:0043230 | extracellular organelle | 1.05E-01 | 2.08 | 20 |
| CC | GO:1903561 | extracellular vesicle | 9.77E-02 | 2.08 | 20 |
| MF | GO:0005201 | extracellular matrix structural constituent | 3.13E-05 | 13.77 | 9 |
| MF | GO:0005198 | structural molecule activity | 4.46E-02 | 4.91 | 10 |
| MF | GO:0005178 | integrin binding | 4.12E-02 | 9.18 | 6 |
| MF | GO:0061134 | peptidase regulator activity | 5.77E-02 | 8.24 | 6 |
| MF | GO:0005518 | collagen binding | 1.39E-01 | 12.82 | 4 |
