# Supplementary material for: Evolution, Expression Patterns, and Distribution of Novel Ribbon Worm Predatory and Defensive Toxins
Source: Mol Biol Evol. 2022 May 5;39(5):msac096. doi: 10.1093/molbev/msac096 (PMC9132205; doi:10.1093/molbev/msac096)

**Supplementary Material online**

Verdes et al 2022. Expression patterns and distribution of novel predatory and defensive toxins in the hoplonemertean Antarctonemertes valida (Nemertea).

**Supplementary Figure 1.** Transversal tissue sections of the *Antarctonemertes valida* proboscis used to generate MALDI-Imaging Mass Spectrometry color maps showing peptide distribution of selected putative predatory toxins.

**
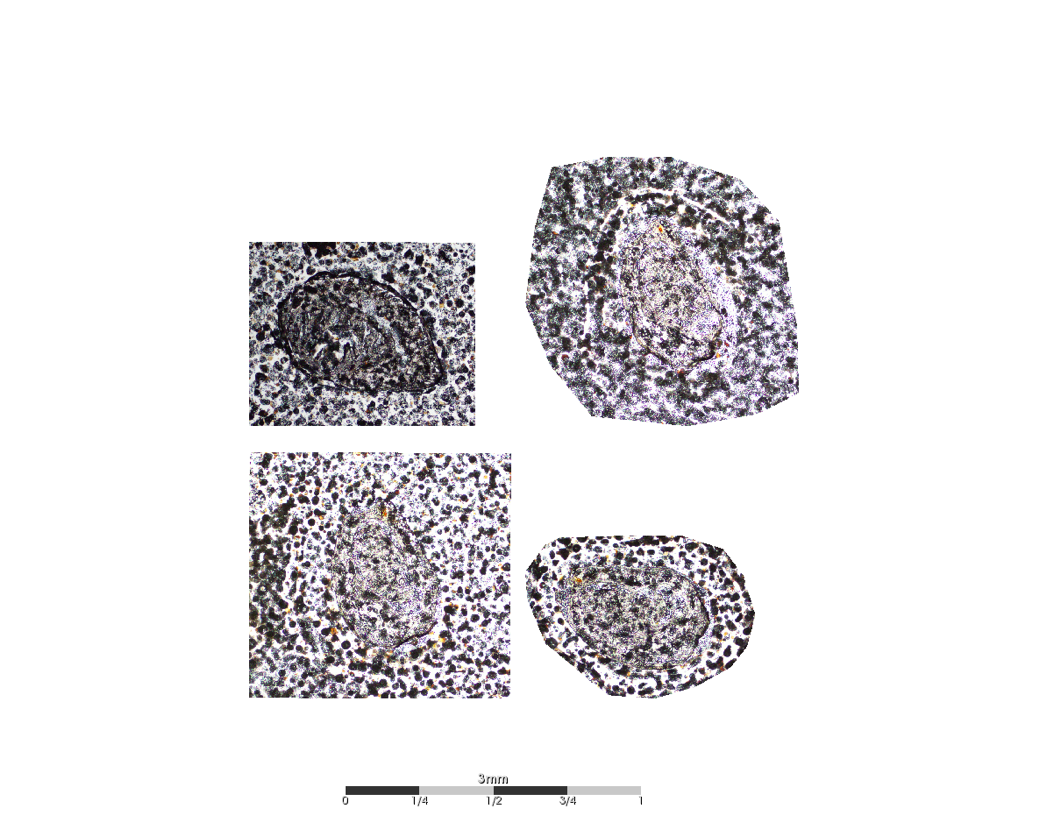
**

**Supplementary Figure 2**. MALDI-IMS color map showing tissue distribution of putative predatory toxin containing insulin-like growth factor binding protein (TRINITY_DN150320_c2_g18_i2).


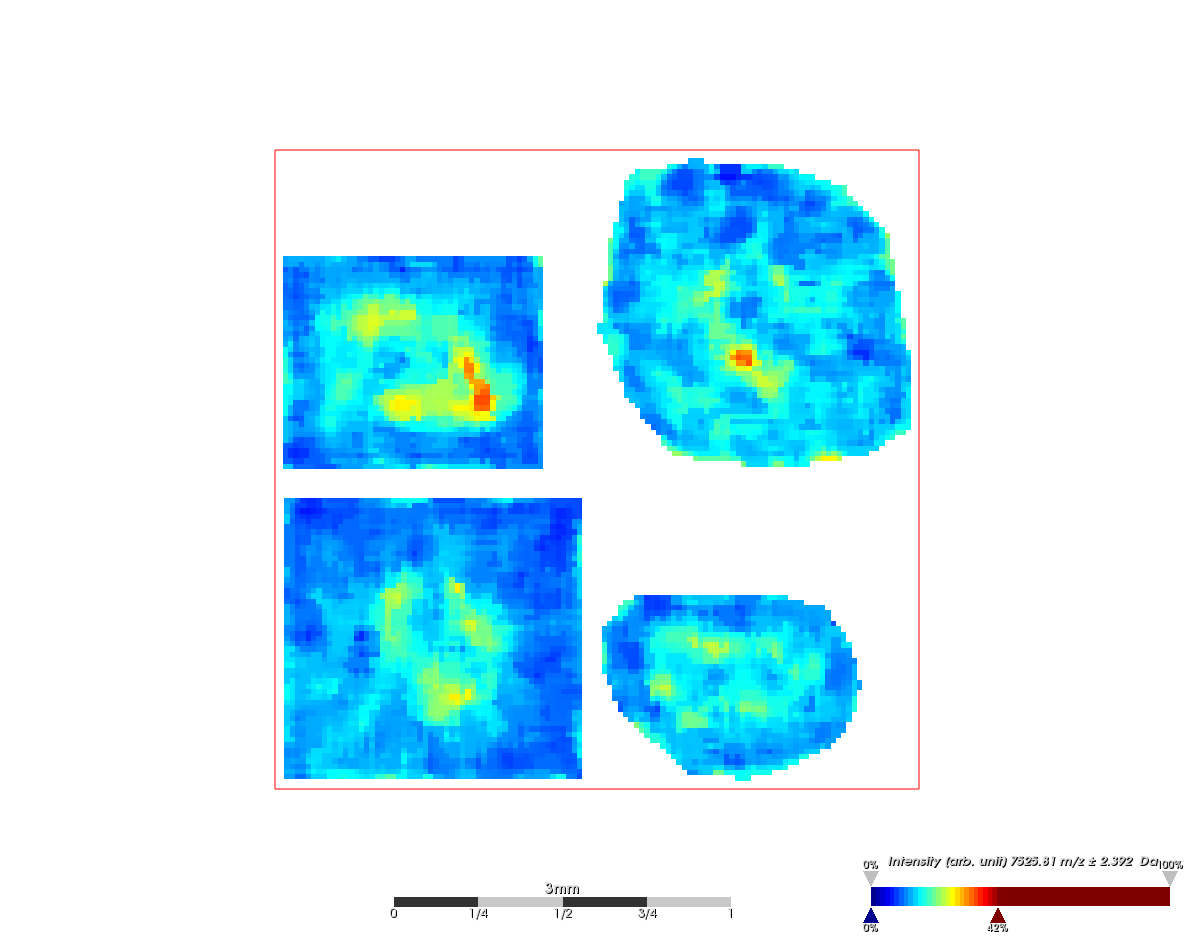


**Supplementary Figure 3**. MALDI-IMS color map showing tissue distribution of putative predatory toxin containing insulin-like growth factor binding protein (TRINITY_DN150320_c2_g18_i4).

**
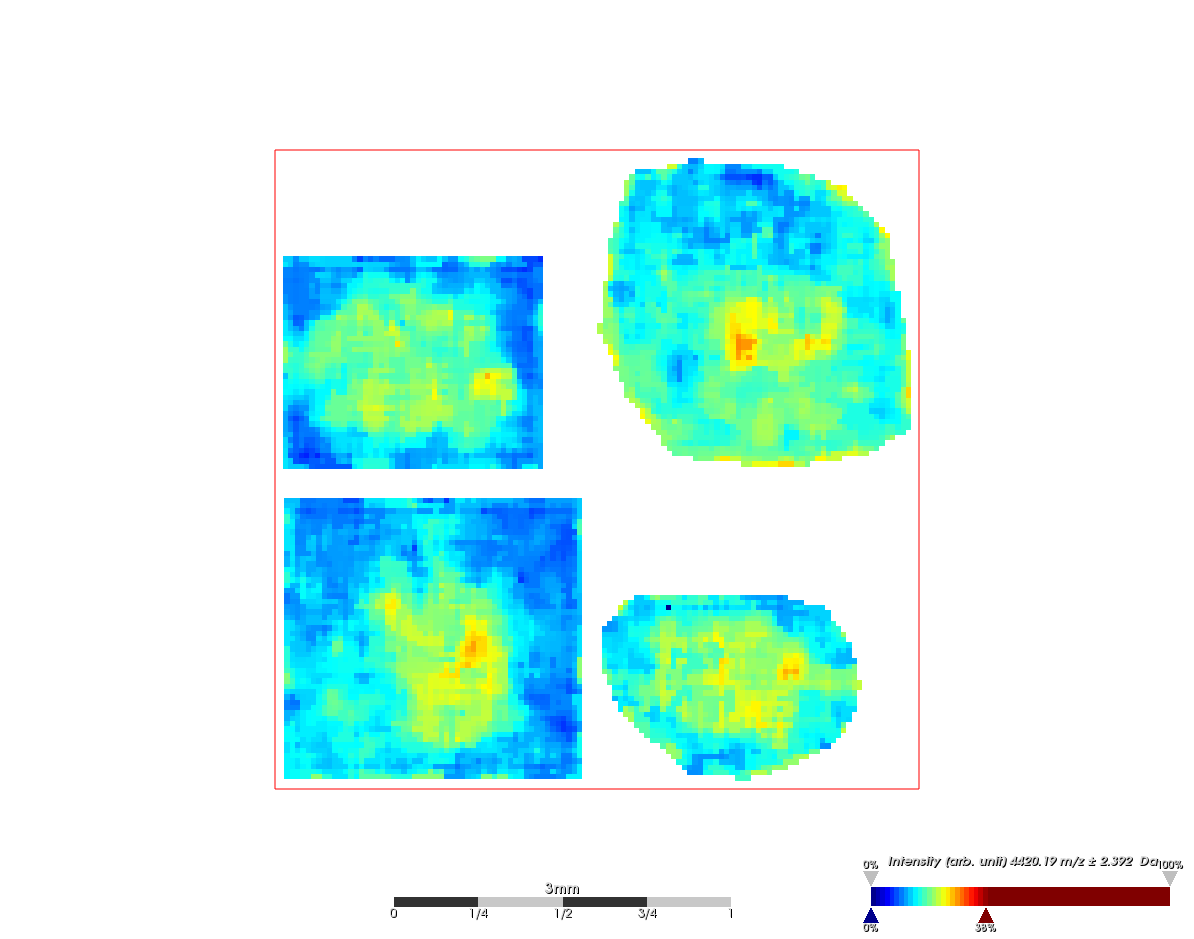
**

**Supplementary Figure 4**. MALDI-IMS color map showing tissue distribution of putative predatory toxin containing insulin-like growth factor binding protein (TRINITY_DN150320_c2_g18_i5).

**
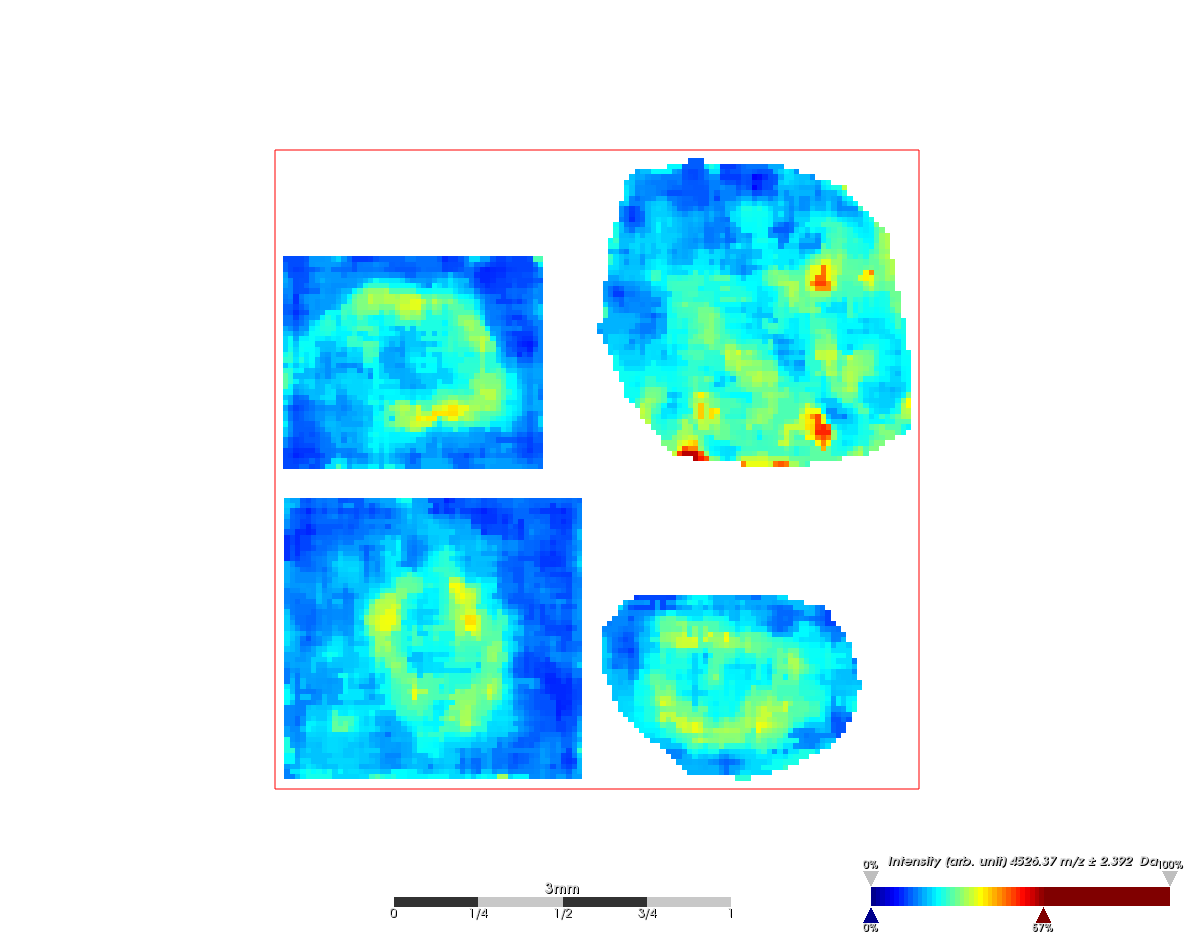
**

**Supplementary Figure 5**. MALDI-IMS color map showing tissue distribution of putative predatory toxin containing insulin-like growth factor binding protein (TRINITY_DN152942_c0_g2_i10).

**
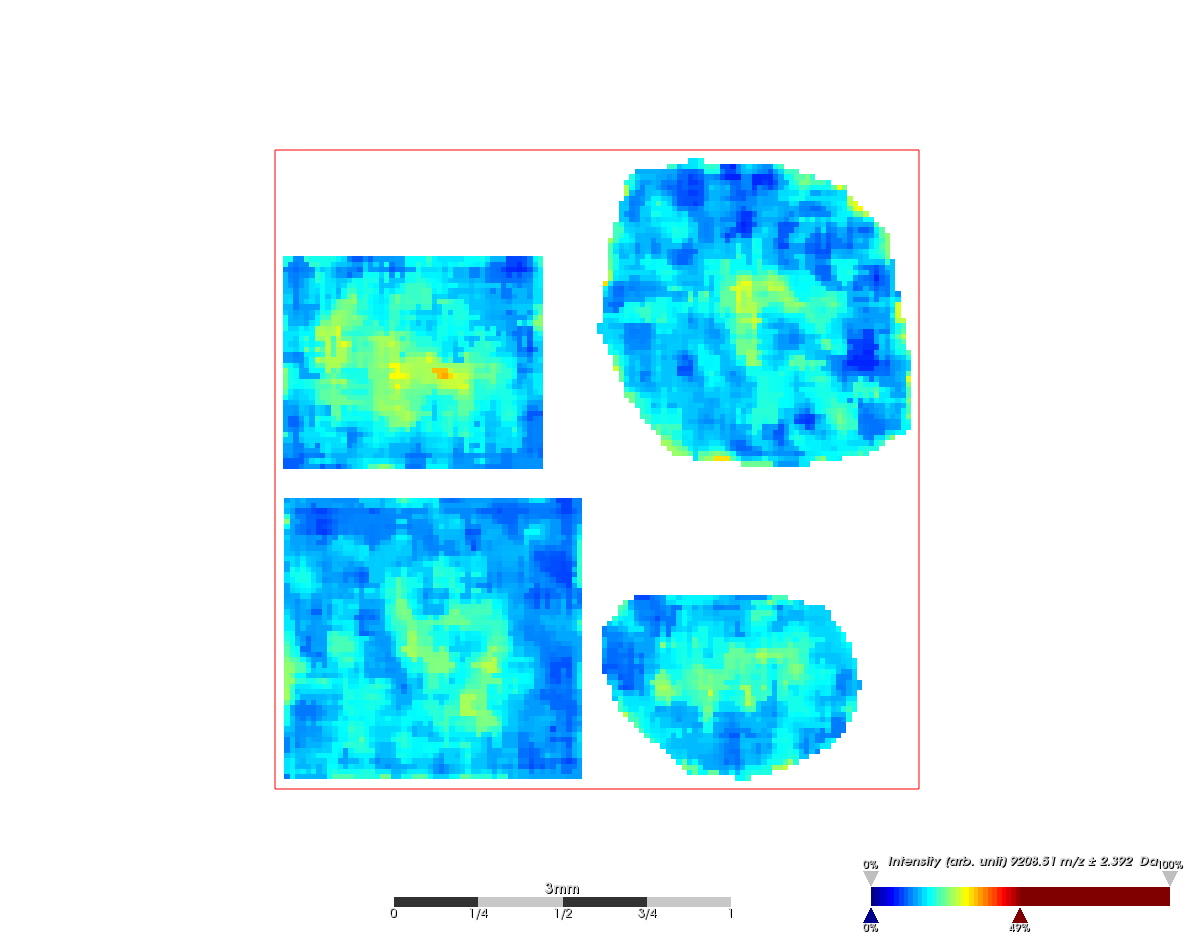
**

**Supplementary Figure 6**. MALDI-IMS color map showing tissue distribution of putative predatory toxin containing galactose binding-like domain (TRINITY_DN150244_c0_g1_i1).


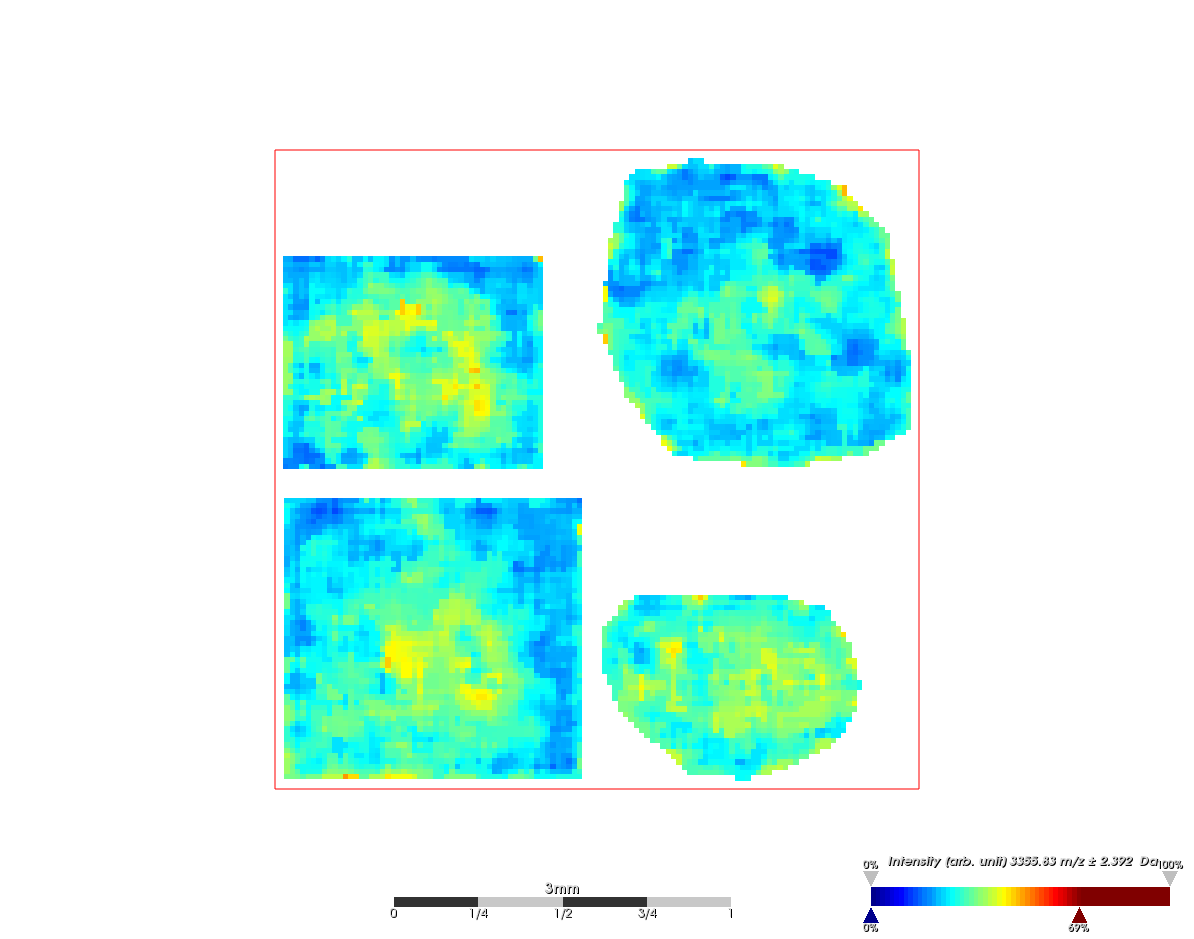


**Supplementary Figure 7**. MALDI-IMS color map showing tissue distribution of putative predatory toxin containing antistasin-like domains (TRINITY_DN140325_c0_g1_i3).


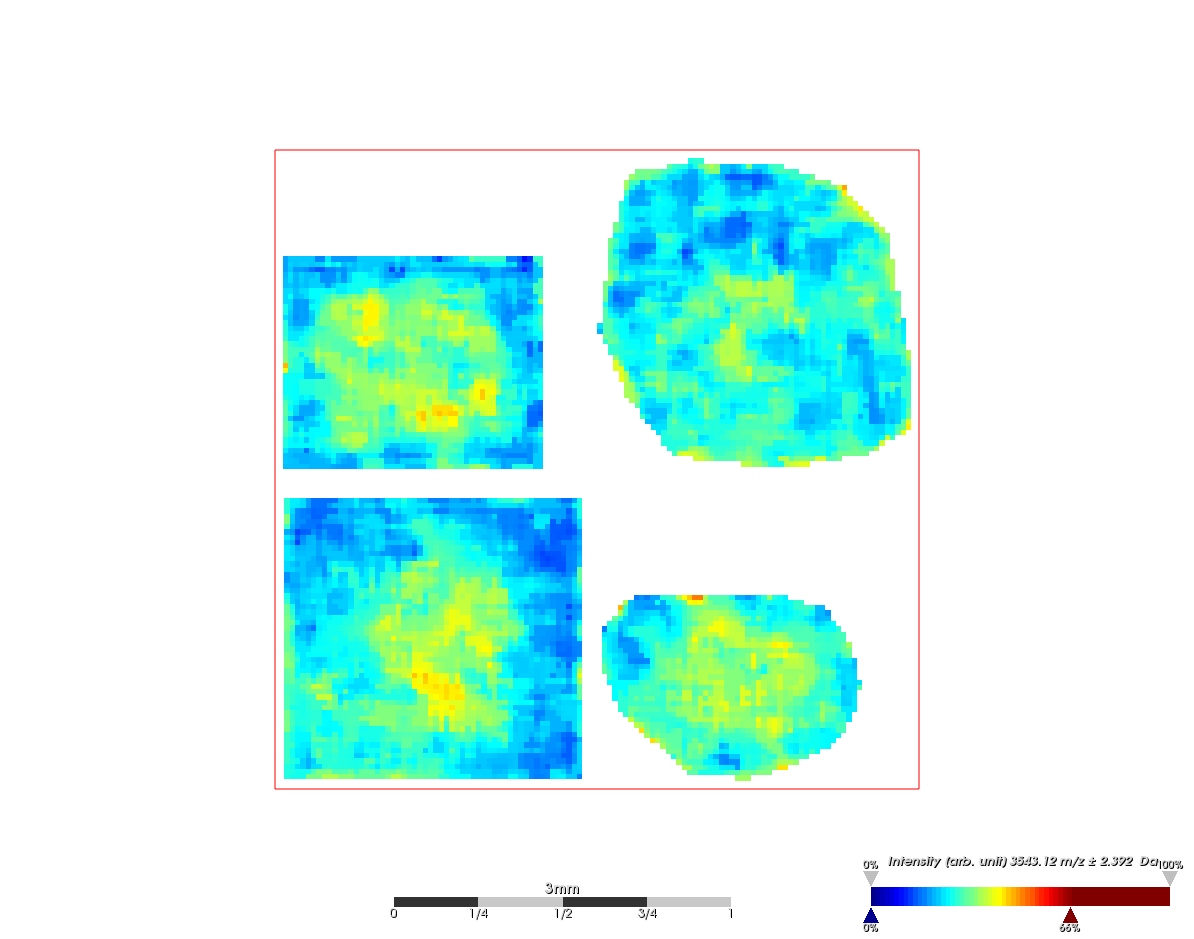


**Supplementary Figure 8**. MALDI-IMS color map showing tissue distribution of putative predatory toxin TRINITY_DN142046_c2_g1_i2.

**
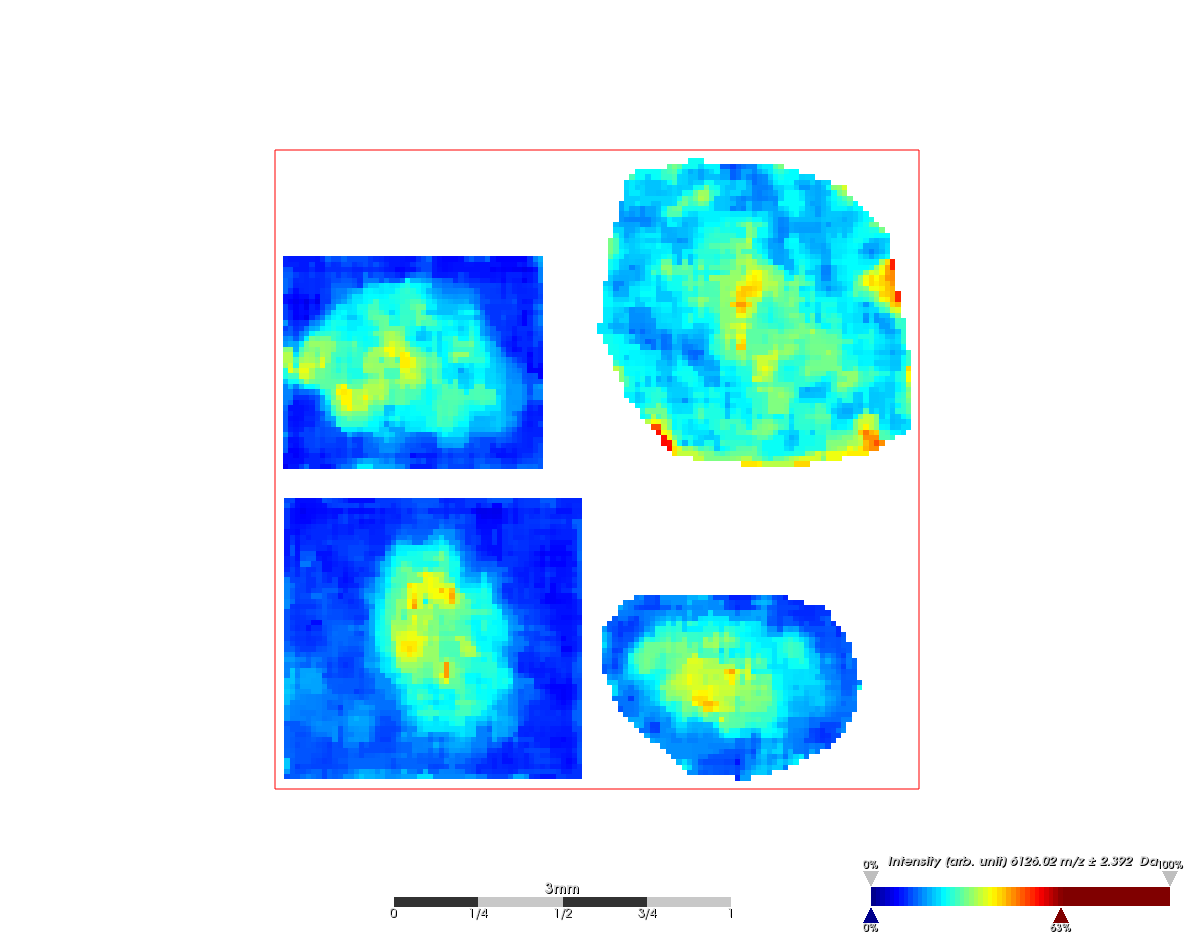
**

**Supplementary Figure 9**. MALDI-IMS color map showing tissue distribution of putative predatory toxin TRINITY_DN135024_c0_g3_i1.


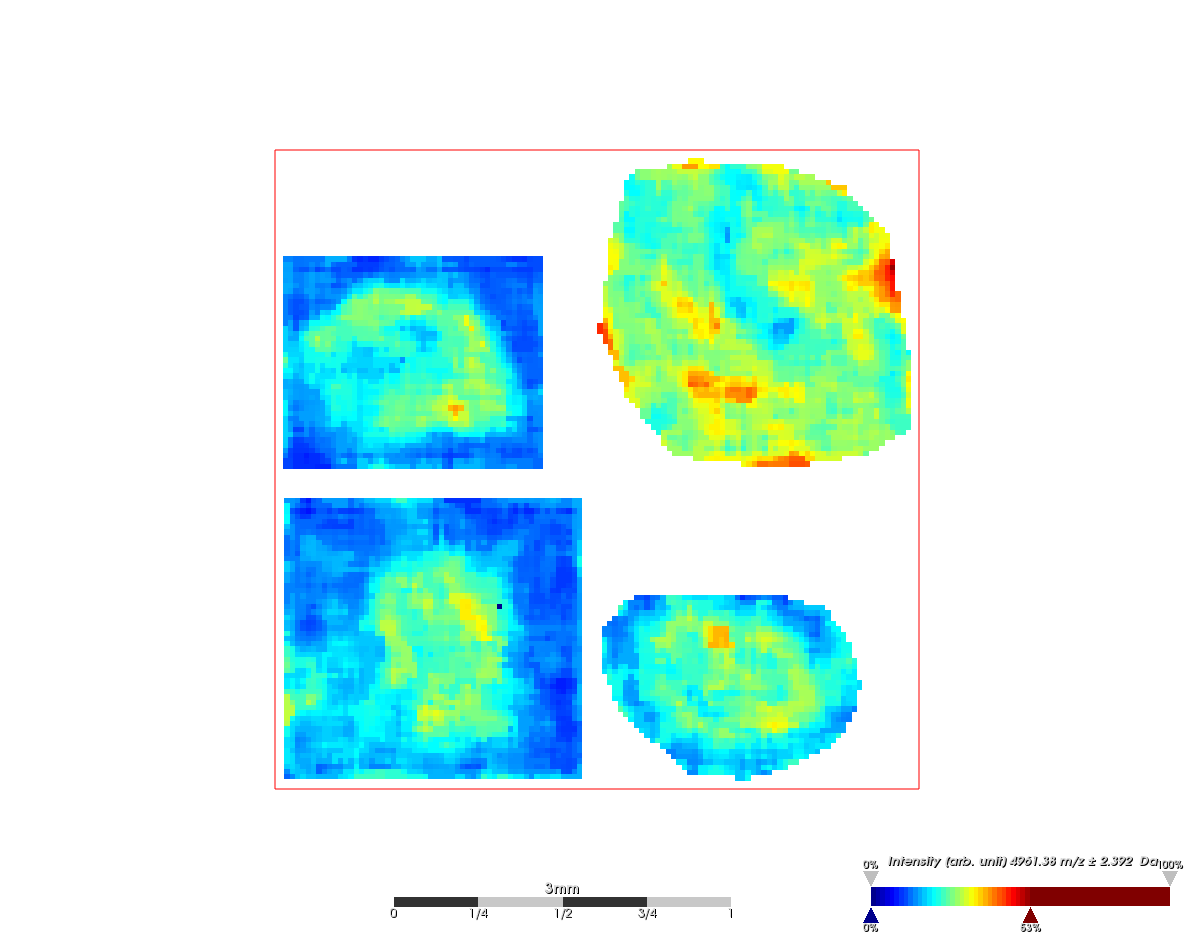


**Supplementary Figure 10.** MALDI-IMS color map showing tissue distribution of putative predatory toxin TRINITY_DN149554_c14_g4_i1.

**
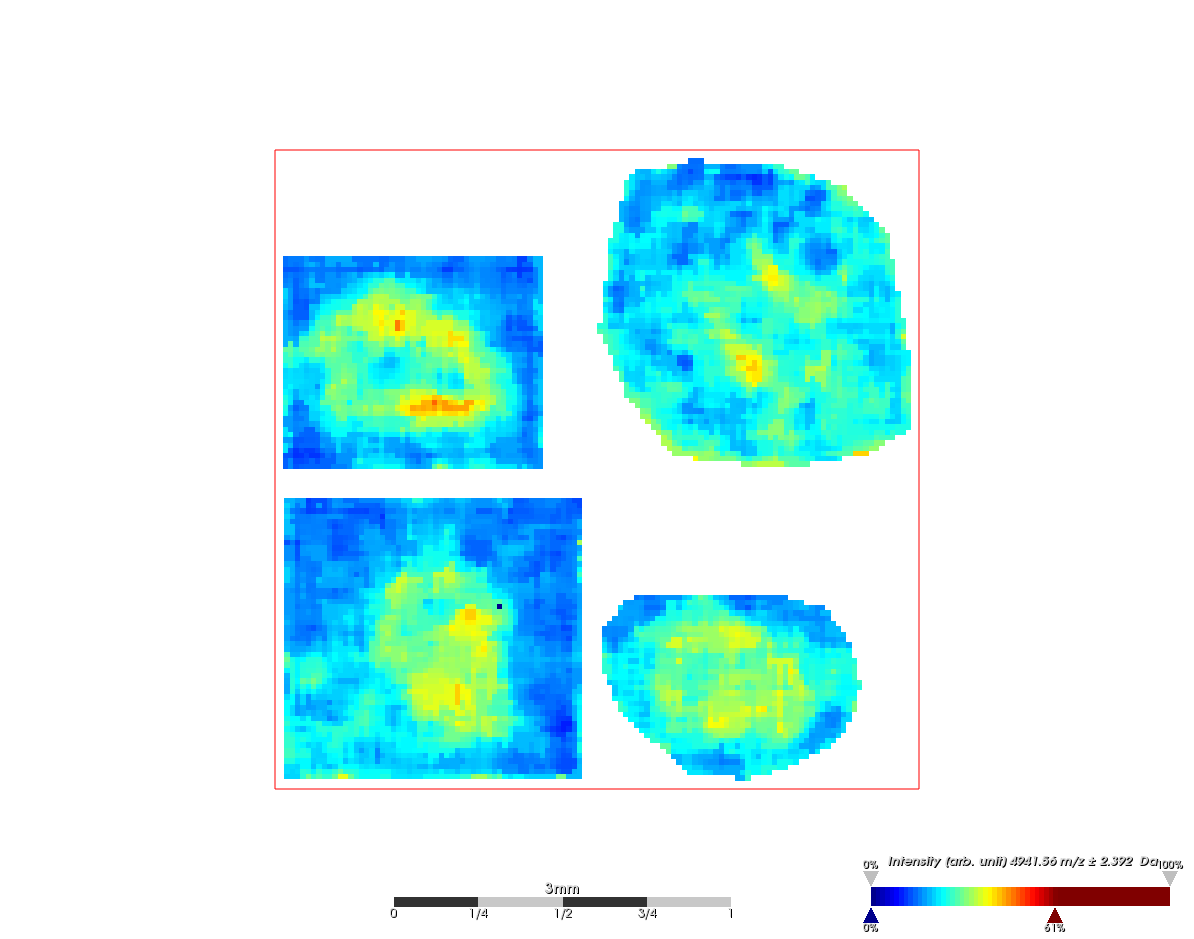
**

**Supplementary Figure 11.** MALDI-IMS color map showing tissue distribution of putative predatory toxin TRINITY_DN149690_c2_g1_i3.

**
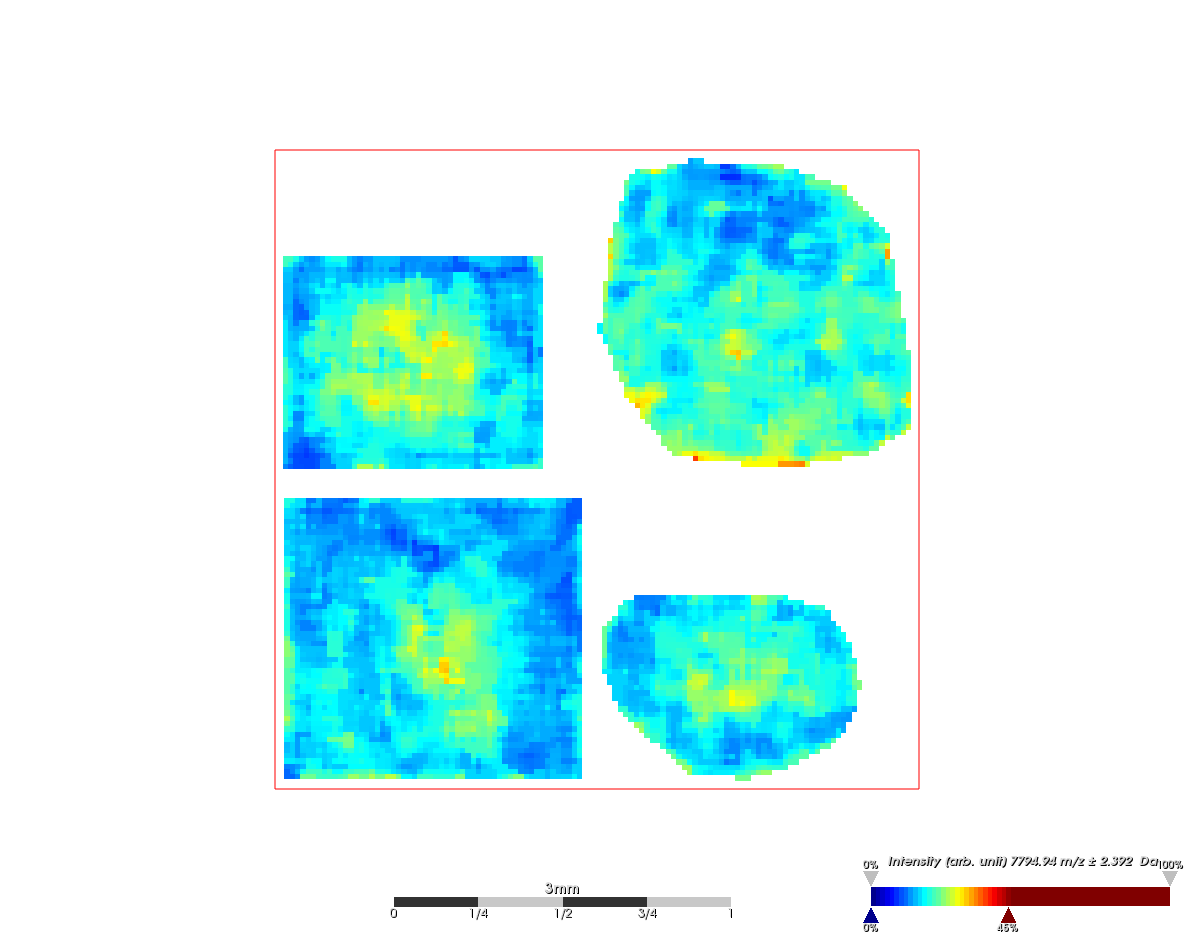
**

**Supplementary Figure 12**. Tissue distribution of unidentified peptide with intense signals and distributions restricted to the glandular inner proboscis epithelium.


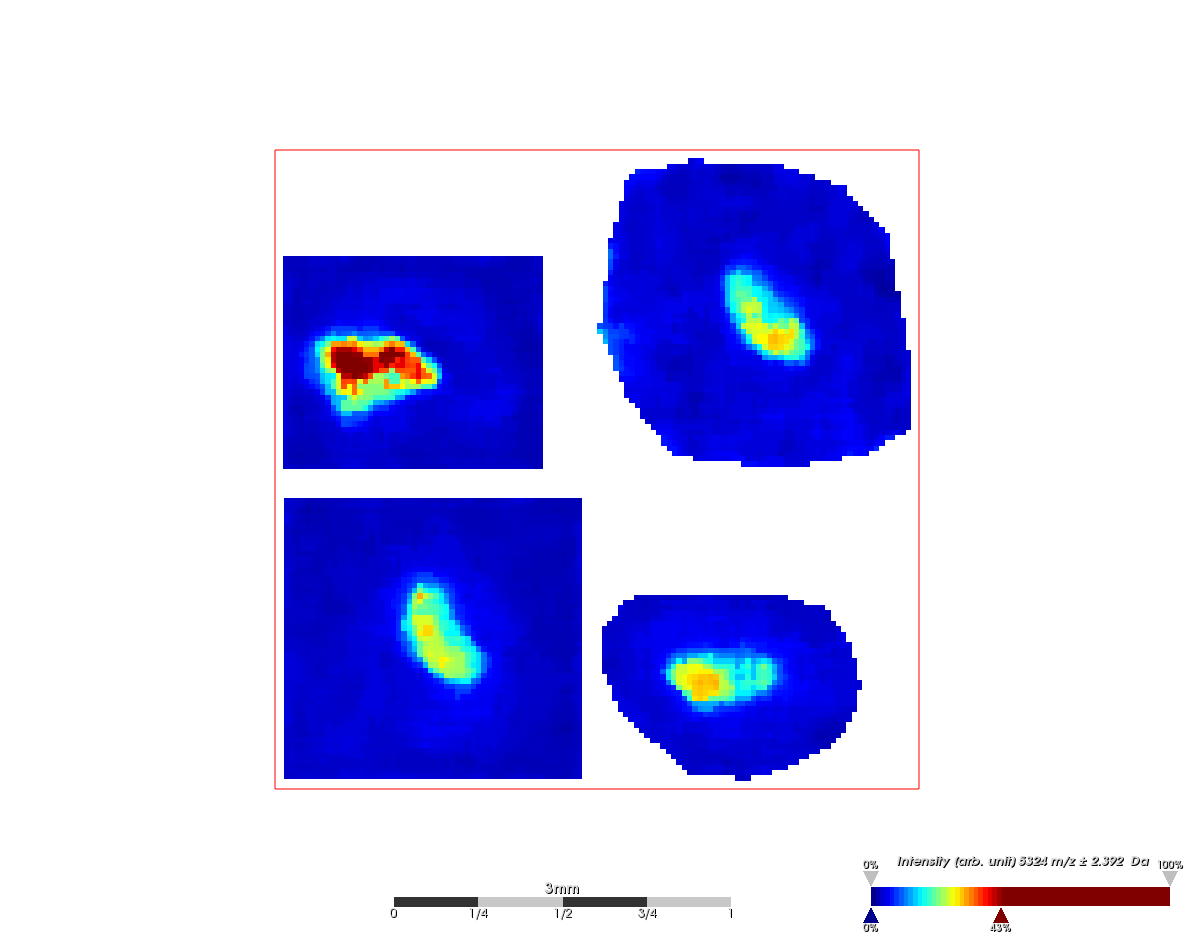

Supplement: msac096_Supplementary_Data [file msac096_supplementary_data.zip › Supplementary_file5._MSI.docx]
